# Disentangling Immediate Adaptive Introgression from Selection on Standing Introgressed Variation in Humans

**DOI:** 10.1093/molbev/msx314

**Published:** 2017-12-06

**Authors:** Evelyn Jagoda, Daniel J Lawson, Jeffrey D Wall, David Lambert, Craig Muller, Michael Westaway, Matthew Leavesley, Terence D Capellini, Marta Mirazón Lahr, Pascale Gerbault, Mark G Thomas, Andrea Bamberg Migliano, Eske Willerslev, Mait Metspalu, Luca Pagani

**Affiliations:** 1 Human Evolutionary Biology, Harvard University, Cambridge, MA; 2 Integrative Epidemiology Unit, Population Health Sciences, University of Bristol, Bristol, United Kingdom; 3 Institute for Human Genetics, University of California, San Francisco, CA; 4 Australian Research Centre for Human Evolution, Environmental Futures Research Institute, Griffith University, Nathan, QLD, Australia; 5 Center for GeoGenetics, University of Copenhagen, Copenhagen, Denmark; 6 Department of Anthropology and Sociology, University of Papua New Guinea, Port Moresby, Papua New Guinea; 7 Tropical Archaeology Research Laboratory, College for Education, Arts and Social Sciences, James Cook University, Cairns, Queensland, Australia; 8 Department of Archaeology, Leverhulme Centre for Human Evolutionary Studies, University of Cambridge, Cambridge, United Kingdom; 9 Research Department of Genetics Evolution and Environment, University College London, London, United Kingdom; 10 Department of Anthropology, University College London, London, United Kingdom; 11 UCL Genetics Institute, University College London, London, United Kingdom; 12 Department of Zoology, University of Cambridge, Cambridge, United Kingdom; 13 Wellcome Trust Sanger Institute, Cambridge, United Kingdom; 14 Estonian Biocentre, Tartu, Estonia; 15 APE Lab, Department of Biology, University of Padova, Padova, Italy

**Keywords:** adaptive introgression, Neanderthal, positive selection, archaic genomes, interferon, toll-like receptor

## Abstract

Recent studies have reported evidence suggesting that portions of contemporary human genomes introgressed from archaic hominin populations went to high frequencies due to positive selection. However, no study to date has specifically addressed the postintrogression population dynamics of these putative cases of adaptive introgression. Here, for the first time, we specifically define cases of immediate adaptive introgression (iAI) in which archaic haplotypes rose to high frequencies in humans as a result of a selective sweep that occurred shortly after the introgression event. We define these cases as distinct from instances of selection on standing introgressed variation (SI), in which an introgressed haplotype initially segregated neutrally and subsequently underwent positive selection. Using a geographically diverse data set, we report novel cases of selection on introgressed variation in living humans and shortlist among these cases those whose selective sweeps are more consistent with having been the product of iAI rather than SI. Many of these novel inferred iAI haplotypes have potential biological relevance, including three that contain immune-related genes in West Siberians, South Asians, and West Eurasians. Overall, our results suggest that iAI may not represent the full picture of positive selection on archaically introgressed haplotypes in humans and that more work needs to be done to analyze the role of SI in the archaic introgression landscape of living humans.

## Introduction

Since the publication of the draft sequence of the first Neanderthal genome (Green et al. 2010), studies consistently report evidence suggesting that the history of modern humans included interbreeding events with now-extinct hominin populations (Skoglund and Jakobsson 2011; Meyer et al. 2012; Prüfer et al. 2014). This interbreeding is reflected in the fact that 1–2% of the genomes of all contemporary non-Africans have a Neanderthal origin (Prüfer et al. 2014) and that ∼3% of Melanesian genomes (Reich et al. 2010, 2011), ∼0.2% of East Asian genomes (Skoglund and Jakobsson 2011), and up to 0.1% of South Asian genomes (Sankararaman et al. 2016) trace their ancestry to Denisovans. Efforts to map these introgressed portions of contemporary human genomes all found that the distribution is not uniform (Sankararaman et al. 2014, 2016; Vernot and Akey 2014; Racimo et al. 2017). While some genomic regions are highly enriched for archaic introgression, others show a depletion.

Natural selection has been invoked to explain the uneven distribution of archaic introgression throughout contemporary human genomes. Regions that have a depletion of archaic ancestry have been suggested to have been subject to negative selection against archaic haplotypes. Conversely, regions enriched for introgression are hypothesized to reflect positive selection on introgressed haplotypes, a phenomenon referred to as “adaptive introgression” (for example Sankararaman et al. 2014). Several studies have provided evidence of adaptive introgression at loci related to the immune system (Dannemann et al. 2016; Abi-Rached et al. 2011; Deschamps et al. 2016; Mendez et al. 2013), lipid metabolism (Khrameeva et al. 2014), keratin filaments (Sankararaman et al. 2014), and high altitude adaptation (Huerta-Sánchez et al. 2014).

No study to date, however, has specifically addressed the post-introgression population dynamics of these putative cases of adaptive introgression in humans. Particularly, the timescale on which positive selection may have acted in cases of putative adaptive introgression is unclear. The narrative typically applied to adaptive introgression in humans is that by interbreeding with archaic hominins, who had been living in and adapting to Eurasian environments for millennia, modern humans dispersing out of Africa acquired useful genetic variants that helped them survive in these new environmental contexts.

### But When Did Selection on These Archaic Haplotypes Act?

Adaptive introgression may occur on two distinct timescales. In the more simple scenario, positive selection on introgressed variation occurs soon after interbreeding and in the population that experienced the gene flow. Here, we refer to these as instances of immediate adaptive introgression (iAI). Alternatively, introgressed haplotypes may undergo an initial period of neutral segregation in the population in which the introgression occurred and only become adaptive at a later period, due to a change in selection pressures acting on the population. This scenario is largely akin to selection on standing variation, except that the source of the variation is introgression. Therefore, here we refer to events following this pattern of more recent selection as instances of selection on standing introgressed variation (SI).

Both iAI and SI could explain the presence of high frequency Neanderthal haplotypes in living human populations. If positive selection acted on introgressed haplotypes in the population dispersing out of Africa soon after interbreeding with Neanderthals and prior to the differentiation of the Eurasian populations, this would be an instance of iAI. However, SI may also have occurred if Neanderthal-introgressed haplotypes initially persisted in modern humans segregating approximately neutrally, despite overall low levels of purifying selection against archaic introgression (Sankararaman et al. 2014; Harris and Nielsen 2016; Juric et al. 2016), potentially because of low effective population sizes. Then, following within-Eurasia population differentiation, some humans would have been exposed to new environments in which these haplotypes became beneficial, leading to positive selection on them. In some instances, these SI sweeps may have been driven by a de novo mutation along an otherwise introgressed haplotype. In this study, however, we are unable to distinguish these latter cases from selection driven solely by introgressed variation, due to the known divergence between the introgressing Neanderthal population and the available high-coverage Altai Neanderthal genome (Prüfer et al. 2014), which may account for those de novo mutations.

Although the primary difference separating iAI and SI is the timescale on which the selection event occurred, another necessary, but not sufficient, feature that may, in principle help to distinguish SI from iAI is the geographic localization of the signal of selection. Following our definition and assuming a model in which Neanderthal introgression was rare and occurred predominantly during the beginning of the Out of Africa dispersal (for example, Green et al. 2010), a given iAI haplotype would be expected to be shared by most Eurasian populations. Conversely, the positive selection events leading to SI likely occurred in geographically localized and genetically differentiated populations. However, even in an iAI scenario, differential selection or random drift acting on an iAI haplotype after an initial period of widespread positive selection may result in a patchier sharing of such signals than would be expected a priori. In the particular example of Neanderthal introgression into modern humans, serial founder events and population expansion during the Out of Africa dispersal could have contributed to the loss, in some descendant populations, of some previously positively-selected introgressed variation. As a result, some genuine iAI haplotypes may exist at high frequency today in only a subset of Eurasian populations. Therefore, the current geographic distribution of a signal of selection on an introgressed haplotype cannot be used as an effective criterion for distinguishing iAI and SI. A more direct measure of the timescale(s) of the selective event is required.

Distinguishing between iAI and SI is highly relevant because many well-documented cases of positive selection on introgressed variation in modern humans are only displayed by one or a few closely related populations. They therefore fall into the ambiguous category of either iAI with complicated subsequent selective dynamics causing the iAI signal to be lost in many populations, or SI. For example, the Denisovan-introgressed *EPAS1* haplotype found in Tibetans (Huerta-Sánchez et al. 2014), shows a signal of positive selection in a particular localized population. Furthermore, the locus containing the *toll-like receptor (TLR) 1–10* cluster, which has also been reported as a case of adaptive introgression in modern humans (Dannemann et al. 2016; Deschamps et al. 2016), shows evidence of independent selective events occurring on seven divergent introgressed haplotypes at this locus in differentiated Eurasian populations. Despite the convincing evidence for the presence of these high frequency, beneficial introgressed haplotypes at both loci, the localized nature of the selection signals (in Tibetans or particular European and East Asian populations, respectively), warrants an investigation into the timescale of the selective sweeps on these haplotypes. Understanding the timescale on which selection occurred on these haplotypes has important implications for understanding the selective pressures leading to the sweep on these haplotypes, and ultimately, the adaptive phenotype driven by these haplotypes.

Here, we present novel definitions of two types of positive selection on introgressed haplotypes in modern humans: iAI and selection on SI. We combined currently existing methods to detect putative positively selected introgressed haplotypes (Racimo et al. 2017), and to infer ancient selective sweeps (Racimo 2015) and apply them on a cross-continental high coverage data set (EGDP; Pagani et al. 2016) to conduct a fine-grained localization of archaically introgressed and positively selected haplotypes geographically and temporally. We specifically shortlisted introgressed haplotypes that experienced a selective sweep prior to the genetic separation of Eastern and Western Eurasians, consistent with having been a result of iAI following our definition (“inferred iAI”) rather than SI or other types of dynamics (oAI, for “other AI”).

## Results

**Fig. 1:**
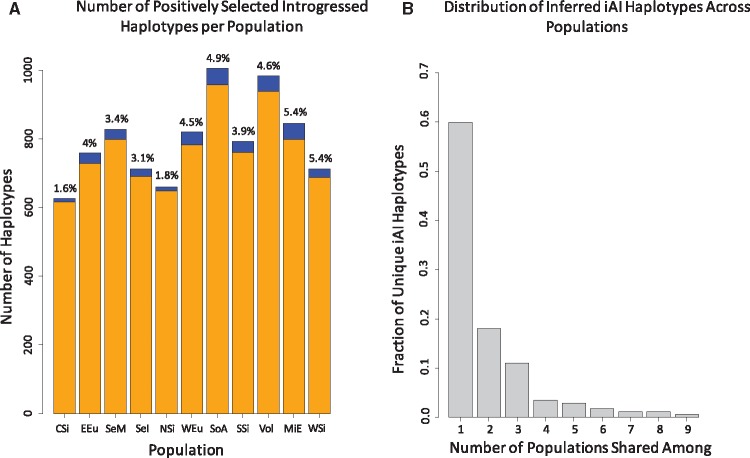
Distribution of inferred iAI and oAI haplotypes in the data set. (*A*) The number of all candidate introgressed and positively selected haplotypes per population. The blue portion of the bar shows the fraction of such haplotypes that were identified as inferred iAI candidates with the remaining fraction of other AI, or oAI, displayed in orange. The percentage of inferred iAI haplotypes is listed above each bar. Population abbreviations are as follows: CSi-Central Siberia; EEu-East and North Europe; SeM-East and Southeast Asia Mainland; SeI-Island Southeast Asia; NSi-Northeast Siberia; WEu-South and West Europe; SoA-South Asia; SSi-South Siberia and Mongolia; Vol-Volga and Ural; MiE-West Asia and Armenia; WSi-Western Siberia. (*B*) Distribution of inferred iAI haplotypes across populations. Haplotypes are binned according to the number of populations they are detected in.

We used a combined approach to identify a number of candidate selection signals on introgressed haplotypes using U20 and Q95 statistics (Racimo et al. 2017), and further shortlisted a conservative list of inferred iAI candidates from this pool using the Three Population Composite Likelihood Ratio (3P-CLR) (Racimo 2015, see Materials and Methods section). For both the Neanderthal-specific and nonspecific archaic introgression U20 and Q95 runs, we identified candidate introgressed haplotypes that have been under positive selection in each of the 11 populations, totaling 3,799 unique 40 kb regions (supplementary table S1, Supplementary Material online). Of these 3,799 detected windows, 3,254 (85.6%) are novel. Half (49.5%) of all windows are detected only in a single population. The joint distributions of the U20 and Q95 statistics for each population in each analysis are shown in supplementary figures S1–S22, Supplementary Material online. We were further able to use our 3P-CLR data to shortlist among these candidates those that were under positive selection in Eurasians prior to the split between East Asians and Europeans, allowing us to identify the most likely haplotypes to represent iAI events rather than events potentially reflecting SI or other selective mechanisms (oAI, supplementary table S2, Supplementary Material online). We were currently unable, however, to estimate the potential presence of true iAI signals within the hits we labeled as oAI. As shown in figure 1*A*, our inferred iAI candidates represent a small fraction of the total number of introgressed and selected haplotypes detected in each population, reaching maximally 5.4% of haplotypes in the Middle East and just 1.6% in the Central Siberian population. An enrichment analysis found that windows most likely to be under ancient selection are not enriched for introgression in any population (see supplementary fig. S23, Supplementary Material online). However, the same windows are enriched for the presence of non-archaic haplotypes (Fisher’s combined *P*-value *P* = 1e-10, supplementary fig. S24, Supplementary Material online); hence, positive selection on the shared Eurasian branch is detected more frequently on human rather than archaic variation.

We further analyzed the distribution of the inferred iAI candidate haplotypes across the populations to explore the degree to which these iAI candidates are localized in individual populations or more widespread. As shown in figure 1*B*, the vast majority of iAI candidates (78%) is detected as iAI in just one population. This result suggests that even if these inferred iAI candidates were initially selected in a population ancestral to all Eurasian populations, they do not segregate at high frequency (≥20%) today (see 3P-CLR Method for a description of this process).

**Fig. 2:**
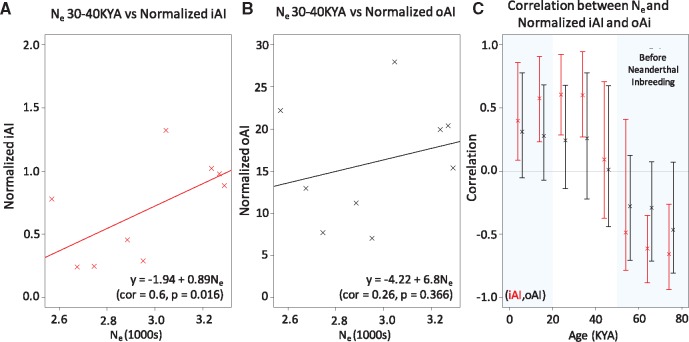
Relationship between effective population size (*N*_e_) and normalized inferred iAI and normalized oAI candidates. For each epoch, the mean *N*_e_ was computed using MSMC by Pagani et al. (2016). The mean *N*_e_ for 30–40 ka is plotted against (*A*) the number of windows detected as inferred iAI candidates in each population normalized by the sample size of the population in our data set and (*B*) the number of oAI candidates also normalized based on sample size. The best fitting linear model, its correlation, as well as the Spearman Rank Correlation test *P*-value is shown. Data for every epoch tested are shown for iAI in supplementary figure S25, Supplementary Material online and for other candidates in supplementary figure S26, Supplementary Material online. (*C*) Raw correlation of normalized iAI and oAI compared with *N*_e_ at different times. The combined *P*-value of the correlation across all time bins is significant with *P* = 0.000051 using Fisher’s method for combining *P*-values. The date of Neanderthal interbreeding is taken as ∼55 ka following Fu et al. (2014). Confidence intervals are calculated by bootstrap resampling.

To explore potential causes for the failure to retain once shared iAI signals in some populations today, we examined the relationship between inferred iAI signals and demography. We found that the number of windows identified as iAI in each population, normalized by sample size, is significantly positively correlated with effective population size (*N*_e_) between 40 and 30 thousand years ago (ka), 30 and 20 ka, 20 and 10 ka (see fig. 2 and supplementary fig. S25, Supplementary Material online). The combined *P*-value of the correlation across all time bins is also significant with *P* = 0.000051, using Fisher’s method for combining *P*-values. The fact that we see such a correlation may indicate that populations with larger effective population sizes were either better able to retain iAI signals over time, or were more likely to experience effective recurrent selection stimuli at these loci, whereas populations with smaller effective population sizes were more likely to lose part of these signals due to genetic drift. The normalized number of oAI haplotypes, those that do not show evidence of selection along the shared Eurasian branch with the 3P-CLR test, detected in each population was not significantly correlated with *N*_e_ across any individual time bin or across all time bins (supplementary fig. S26, Supplementary Material online). However, the trend of the relationship between normalized oAI and *N*_e_ is similar to that of normalized iAI (fig. 2*C*).

**Fig. 3:**
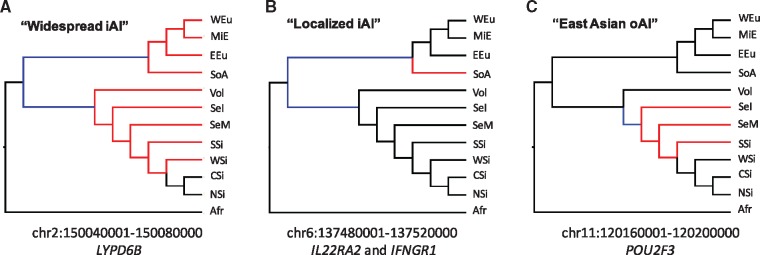
Population relationships of two inferred iAI and one oAI haplotype. We generated a simple Neighbor-Joining population tree based on the average MSMC split time estimates reported in Pagani et al. (2016). Red branches show in which population a (*A*) widespread iAI (chr2: 150040001-150080000 [hg19], *LYD6B*), (*B*) a localized iAI (chr6: 137480001-137520000, *IL22RA2*, and *IFNGR1*), and (*C*) one oAI (chr11: 120160001-120200000, *POU2F3*) case were detected. Blue branches show the hypothetical places where the selective sweeps may have occurred. Population abbreviations are as follows: CSi-Central Siberia; EEu-East and North Europe; SeM-East and Southeast Asia Mainland; SeI-Island Southeast Asia; NSi-Northeast Siberia; WEu-South and West Europe; SoA-South Asia; SSi-South Siberia and Mongolia; Vol-Volga and Ural; MiE-West Asia and Armenia; WSi-Western Siberia.

To compare the population distribution of haplotypes with different demographic histories, we also generated a Neighbor-Joining population tree based on average MSMC split time estimates (from Pagani et al. 2016) and placed on it our most widespread iAI candidate (detected in 9 out of 11 populations), an iAI candidate detected only in one population and an oAI candidate (fig. 3*A*–*C*). Particularly, we highlighted in red the tree branches where a given iAI or oAI signal was detected and in blue the branches where the selective sweep may have taken place.

## Discussion

We were able to detect a number of introgressed haplotypes that may have undergone positive selection. However, we were only able to assign a minor fraction of these selective sweeps to a time period more consistent with iAI than those that reflect SI or other selective mechanisms. Most notably, while replicated in our novel data set of introgressed and potentially adaptive signals, many well-documented high frequency introgressed loci such as *BNC2*, the *TLR1*, *TLR6*, and *TLR10* cluster, and *POU2F3* (see the “previously reported” column of supplementary table S1, Supplementary Material online for the specific references) were not included in our list of inferred iAI candidates. Additionally, we did not find evidence of enrichment for inferred iAI windows in any population, even despite our somewhat relaxed introgression detection statistics. Furthermore, we report how different trends of effective population size changes in various populations may have affected the capability of retaining iAI signals over time. This may, along with putative recurrent selective pressures, explain why some of the currently reported iAI signals are shared only by a limited number of Eurasian populations.

Indeed, among the iAI candidates that we did detect, the majority are not widespread across Eurasian populations. For example, of 172 iAI candidates, only four were detected as iAI in at least 8/11 geographically diverse Eurasian groups, indicating that many iAI haplotypes do not exist at high frequency in individual Eurasian populations today. This may be a result of subsequent purifying selection or random genetic drift that occurred as a result of the serial founder events and simultaneous population expansion following the dispersal out of Africa.

One of the few widely distributed likely iAI haplotypes we detected contains the *LYPD6B* gene. This gene was inferred to have been introgressed from Neanderthals and under positive selection (Racimo et al. 2017). Here, we identify this haplotype as a top iAI candidate and detect it in 9 out of 11 Eurasian populations (fig. 3*A*). *LYPD6B* has been shown to regulate cholinergic signaling in the brain (Arvaniti et al. 2016). Targeted knockouts of this gene in mice show four significant phenotypes: decreased caudal vertebrae number, increase exploration in new environment, increased blood urea nitrogen level, and decreased circulating insulin level (International Knockout Mouse Consortium MGI 2014). These results suggest that selection on this introgressed haplotype may have been due to beneficial behavioral and/or physiological traits, although this requires further functional investigation. Another well known, although less widespread iAI candidate we validated with our approach encompasses a haplotype containing the *OAS2* and *OAS3* genes, both of which are involved in immune function. We detect iAI at this locus in our Middle East, South and West Europe, and East and North Europe populations, supporting previous claims of adaptive introgression at this locus detected in Europeans from the 1,000 Genomes Project (Racimo et al. 2017; Mendez et al. 2013; Gittelman et al. 2016).

Among our novel candidate iAI loci are three haplotypes containing genes related to immune function. One of these haplotypes, containing the *VNN1* gene, is detected only in the West Siberian population, reflecting the additional insights gained by studying previously under-studied populations. The ortholog of this gene in mice has been shown to be important in response to *Coxiella burnetti* infection (Meghari et al. 2007), which leads to Q-fever (Mege et al. 1997). Mice deficient for the orthologous *Vnn1* gene show extreme reduction in granuloma formation in the spleen and liver, which is critical to defense against *C. burnetti* infection (Meghari et al. 2007). Interestingly, *TLR1* and *TLR6*, two genes found to be present on a high frequency introgressed haplotype in this and other studies (Dannemann et al. 2016; Deschamps et al. 2016), have also been shown to play a role in response to *C. burnetti* infection (Ammerdorffer et al. 2015). Another newly detected iAI haplotype is a haplotype detected only in our South Asian population that includes two immunity related genes: *IL22RA2* and *IFNGR1* (fig. 3*B*). *IL22RA2* is involved in the regulation of inflammatory response (Xu et al. 2001), while *IFNGR1* encodes the ligand-binding chain of the interferon receptor (Kotenko et al. 1995). This is consistent with previous reports of innate immunity genes being targets of positive selection on introgressed variation (Dannemann et al. 2016; Deschamps et al. 2016) and specifically with reports of such selection on interferon coding genes in Asian populations (Deschamps et al. 2016). Given that both the *VNN1* containing haplotype in West Siberians and the *IL22RA2/IFNGR1* containing haplotype in South Asians are each detected only in one of our populations, they may represent examples of iAI signals that are localized today likely due to genetic drift compounded by serial founder effects and population expansion during the Out of Africa dispersal, leading to the reduction in frequency of adaptive introgressed haplotypes that underwent initially incomplete iAI sweeps. We also report here for the first time a likely iAI candidate haplotype in several West Eurasian populations, including our newly studied Volga Uralic and West Asia Armenia populations (in addition to South and West Europe and East and North Europe) that includes the *TNFRSF13B* gene. This gene is crucial for terminal B-cell differentiation and therefore variation in this gene and its expression pattern may influence humoral immunity (Romberg et al. 2014). Taken together, these three haplotypes represent examples of Neanderthals contributing immunity-related genetic variation that may have been adaptive to human populations, consistent with similar signals found in 1,000 Genomes population samples (Dannemann et al. 2016; Deschamps et al. 2016; Mendez et al. 2013).

In conclusion, by combining selection and introgression statistics we propose a means to clarify the emerging field of archaic introgression. Particularly, using a systematic scan of a novel data set, we were able to recapitulate many previously reported introgressed haplotypes, to discover 3,254 novel signals and to shortlist among those haplotypes those that are most likely the result of iAI rather than SI or other selective processes. Finally, the fact that the majority of high frequency, introgressed haplotypes were not shortlisted here as inferred iAI candidates—including several gene regions that have been postulated as clear cases of adaptive introgression—confirms that the definition of iAI and SI introduced in the present study is crucial to contextualize the biological meaning of the results reported so far. Further work should be done to understand the role(s) that SI and other selective mechanisms have played in contributing to high frequency archaically introgressed haplotypes. This work should be considered in the context of previously reported evidence of purifying selection as the primary consequence of archaic introgression (Sankararaman et al. 2014; Harris and Nielsen 2016; Juric et al. 2016). If in fact SI was to be found to account for the majority of high frequency introgressed haplotypes, one may speculate that the relative abundance of SI over iAI may simply be the consequence of the longer amount of evolutionary time Eurasian populations spent as differentiated groups. Alternatively, a relative abundance of SI compared with iAI may have been facilitated by a progressive removal of Neanderthal deleterious mutations through recombination, resulting in subsequent availability of adaptive alleles to the human gene pool.

## Materials and Methods

### Sequenced Data

#### Data Set

We grouped the 483 individuals in the recently published Estonian Biocentre Human Genome Diversity Panel (EGDP; Pagani et al. 2016) into 11 macropopulations, as described in the original publication: Central Siberia, Northeast Siberia, West Siberia, South Siberia and Mongolia, West Asia and Armenia, South and West Europe, East and North Europe, Volga and Ural, South Asia, East and Southeast Asia, and Mainland and Island Southeast Asia. We then used the methodology described by Racimo et al. (2017) to search in these populations for introgressed haplotypes that likely experienced positive selection, regardless of the timescale.

#### U20 and Q95 Statistics

To carry out these analyses, we first conducted a full genome scan using a 40 kb window and calculated two statistics for each window, called U (the number of single nucleotide polymorphisms [SNPs] shared with an archaic genome at high frequency in an ingroup population and at low frequency in an outgroup population) and Q95 (the 95% quantile of the frequency of the derived alleles that are shared with an archaic genome and at low frequency in an outgroup population; Racimo et al. 2017). For the *U* statistic, we used the specific form of the statistic *U_AFR, A, Nea_*(1%, 20%, 100%) or “U20,” where *A* is one of the 11 EGDP macropopulations (Pagani et al. 2016), AFR is a combined panel of all the African individuals, with the exception of African Americans, from the 1,000 Genomes Phase 3 data set (The 1000 Genomes Project Consortium 2015), and Nea is the high coverage Altai Neanderthal. Therefore, for each population, the U20 score for each 40 kb window represents the number of SNPs in that window that are at < 1% frequency in the combined African panel, at least 20% frequency in the nonAfrican macropopulation being considered, and 100% frequency (homozygous) in the Altai Neanderthal. We also calculated the Q95 statistic in the form of *Q_A, AFR, Nea_*(1%, 100%) referring to the 95% quantile frequency of derived alleles in one of the macropopulations that are homozygous in the Altai Neanderthal and at < 1% frequency in the combined African panel. 40 kb windows falling within the top 99th percentile in both the U20 and Q95 statistics are considered to be introgressed regions that underwent selection at some point in time. We chose to use this relaxed threshold from the original 99.9th percentile (FDR 0–5.5%) described by Racimo et al. (2017) because of our downstream stringent filtering step to detect ancient selection (see below). Using this approach, we conducted two runs, one in which we conditioned the shared Altai alleles to be different from the Denisovan, and the other without this condition. This allowed us to identify both introgression that is definitively Neanderthal in origin, as well as introgression that is likely Neanderthal in origin, but could also come from another archaic hominin.

#### 3P-CLR Statistic

From this list of introgressed haplotypes that show evidence of having been adaptive, we next sought to identify those haplotypes that underwent positive selection at a time point consistent with iAI rather than SI (i.e., before the major population subdivisions within Eurasia took place). Although already available for this data set (Pagani et al. 2016), tests of recent selection, such as Tajima’s D (Tajima 1989), iHS (Voight et al. 2006), and nSL (Ferrer-Admetlla et al. 2014) cannot provide a reliable timescale of a selective sweep, because the homozygosity signal may persist well after the selective event. We therefore sought to directly search for ancient selection acting on the broader Eurasian branch to shortlist only haplotypes that could be inferred to be iAI. To do so, we used the 3P-CLR statistic (Racimo 2015) to look for regions in the EGDP data set that show evidence of selection that likely occurred shortly after the expansion out of Africa, and hence soon after the purported interbreeding event between Neanderthals and modern humans (Green et al. 2010). The 3P-CLR statistic assumes a three-population tree model with no post-split migration. We ran the 3P-CLR test to look for selection that occurred after the split between Africans and Eurasians in the EGDP data set, but prior to the split between East Asians and Europeans. This timescale of selection would be consistent with the major pulse of Neanderthal introgression into an undifferentiated Eurasian population as suggested by Green et al. (2010). To ensure that the individuals used in the 3P-CLR analyses represent the most basal split within living Eurasian populations, we used for our East Asian population only Chinese and Japanese individuals from the Mainland East and Southeast Asia macropopulation. The European individuals used were a random subset of the South and West Europe and East and North Europe populations. The African outgroup population consisted of the Yoruba individuals from the EGDP data set (Pagani et al. 2016). Following Racimo (2015), 100 SNPs (with at least 20 SNPs between them) were sampled in each window of length 0.5 Morgans.

Upon completion of the scan, sampled SNPs were grouped into 200 kb bins that were assigned the maximum 3P-CLR score of the sampled SNPs in the window. Windows within the top 99th percentile of scores from this 3P-CLR test were considered to be under selection along the shared Eurasian branch. We inferred as iAI only the haplotypes that appeared to be introgressed and under positive selection in at least one Eurasian population (using the U20 and Q95 statistics), and that also showed signs that the selection on them was ancient and is currently detectable in Europeans and East Asians (according to the 3P-CLR test). We note that our filtering procedures should ensure a given inferred iAI haplotype to be present at some frequency throughout Eurasia due to its high-ranking 3P-CLR score. However, should a putative iAI haplotype frequency fall below 20% in any given population, it may be removed by the U20 and Q95 filters and hence result in a genuine iAI signal to be detected only in a subset of modern nonAfrican populations. We also note that, while the hits that pass our criteria can be considered as “inferred iAI,” we cannot currently rule out that the remaining oAI (“other AI”) hits are completely depleted of iAI signals. We therefore interpret our inferred set of iAI haplotypes as a useful yet conservative pool with which to explore iAI in living humans.

### Enrichment Analysis

To test the enrichment of the overlap between the 3P-CLR and U20 and Q95 tests for each population, we conducted a bootstrap analysis with 5,000 replicates, each time randomly assigning 3P-CLR scores to every top 40 kb window based on the U20 and Q95 statistics. A *P*-value was assigned to each threshold of top 3P-CLR windows based on the proportion of bootstrap tests in which the randomized sample had the same or fewer top U20 and Q95 windows overlapping with 3P-CLR windows within the threshold than the observed data. Because we did not know the optimal number of windows to choose in advance, we chose the number that minimized the *P*-value. To account for this, we performed a second level of bootstrap in which we replicated our test statistic calculation by also choosing the best *P*-value for the replicates (restricted to be within the top 800 windows). This yields theoretically conservative *P*-values for enrichment, $p_{i}^{enrich}$; a single *P*-value for each population.

### Effective Population Analysis

Finally, we started from the basic population genetic notion that effective population size (*N*_e_) positively correlates with the efficacy of a selective sweep and, hence, with the ability of a population to retain signature of a past selective sweep (Nielsen and Slatkin 2013). To examine whether our set of inferred iAI signals are associated with effective population size, we explored the correlation between the number of iAI hits per population, normalized by the sample size of the population in our data set, and the harmonic mean *N*_e_ of that population. We conducted the same analysis for the set of oAI haplotypes. We calculated harmonic mean *N*_e_ by averaging over bins of 10 ka from present to 70 ka, starting from the MSMC based (Schiffels and Durbin 2014) *N*_e_ estimates reported in Pagani et al. (2016), using the nine macropopulations where at least four genomes from a given homogeneous population were available. We then fit a linear model to the data allowing an intercept term with the number of normalized iAI or oAI respectively) acting as the response variable and the *N*_e_ of the population at each time bin as the predictor variable. To avoid making distributional assumptions for assessing uncertainty in the trend of iAI/oAI with *N*_e_, we performed bootstrap resampling of the (iAI/oAI, *N*_e_) pairs. This provides a distribution of values for the association by refitting the linear model each time (1,000 resamplings; the 5–95% quantiles are reported if fig. 2*C*). We then report a two-tailed *P*-value (reported in fig. 2*A*, *B*;supplementary figs. S25, S26, Supplementary Material online).

## Supplementary Material

Supplementary data are available at *Molecular Biology and Evolution* online.

## Supplementary Material

Supplementary Data
