# Supplementary material for: Disentangling Immediate Adaptive Introgression from Selection on Standing Introgressed Variation in Humans
Source: Mol Biol Evol. 2017 Dec 6;35(3):623–30. doi: 10.1093/molbev/msx314 (PMC5850494; doi:10.1093/molbev/msx314)

Figure S1

Distribution of U20 and Q95 Statistics Run 1
Central Siberia

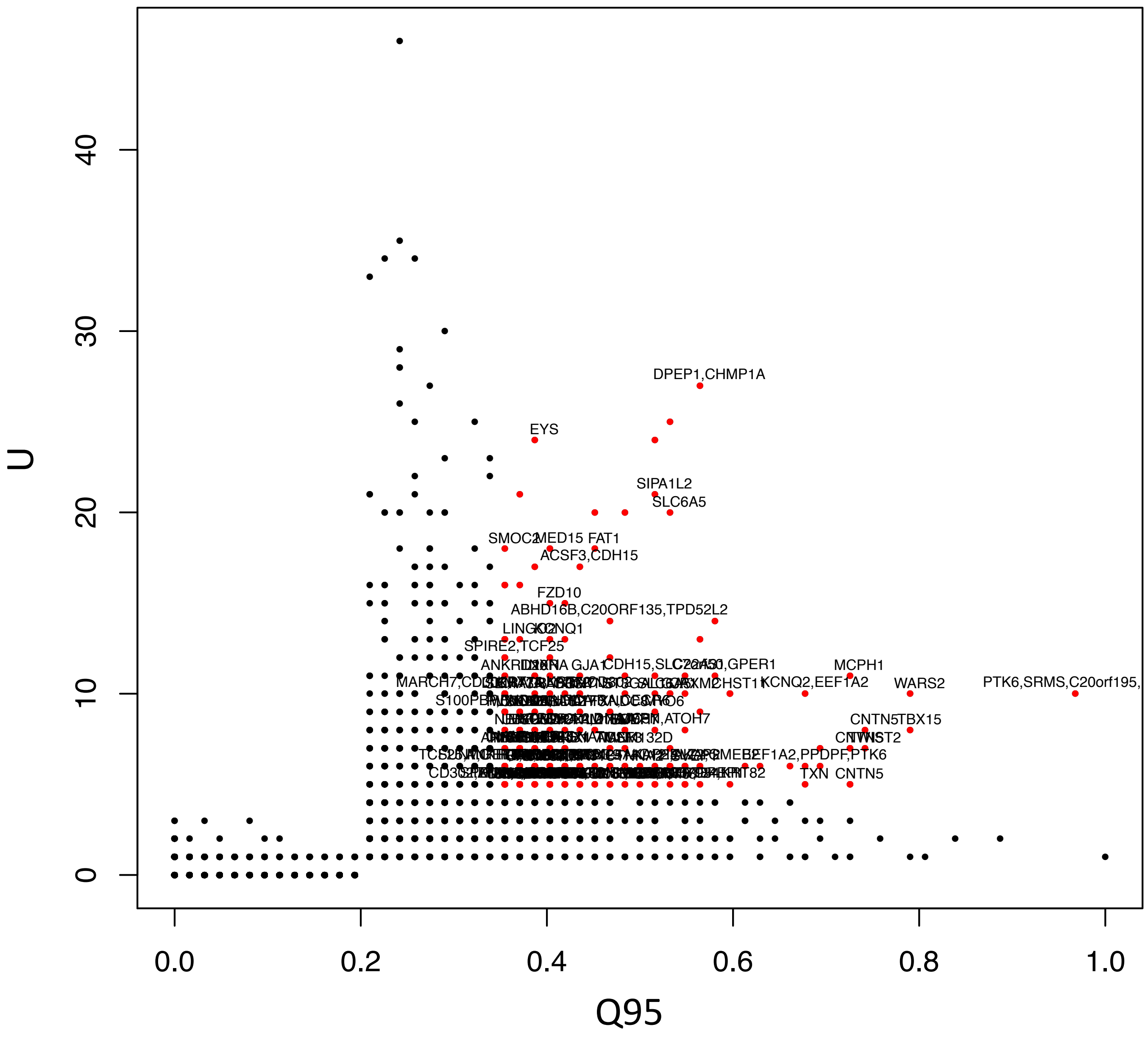

Figure S2

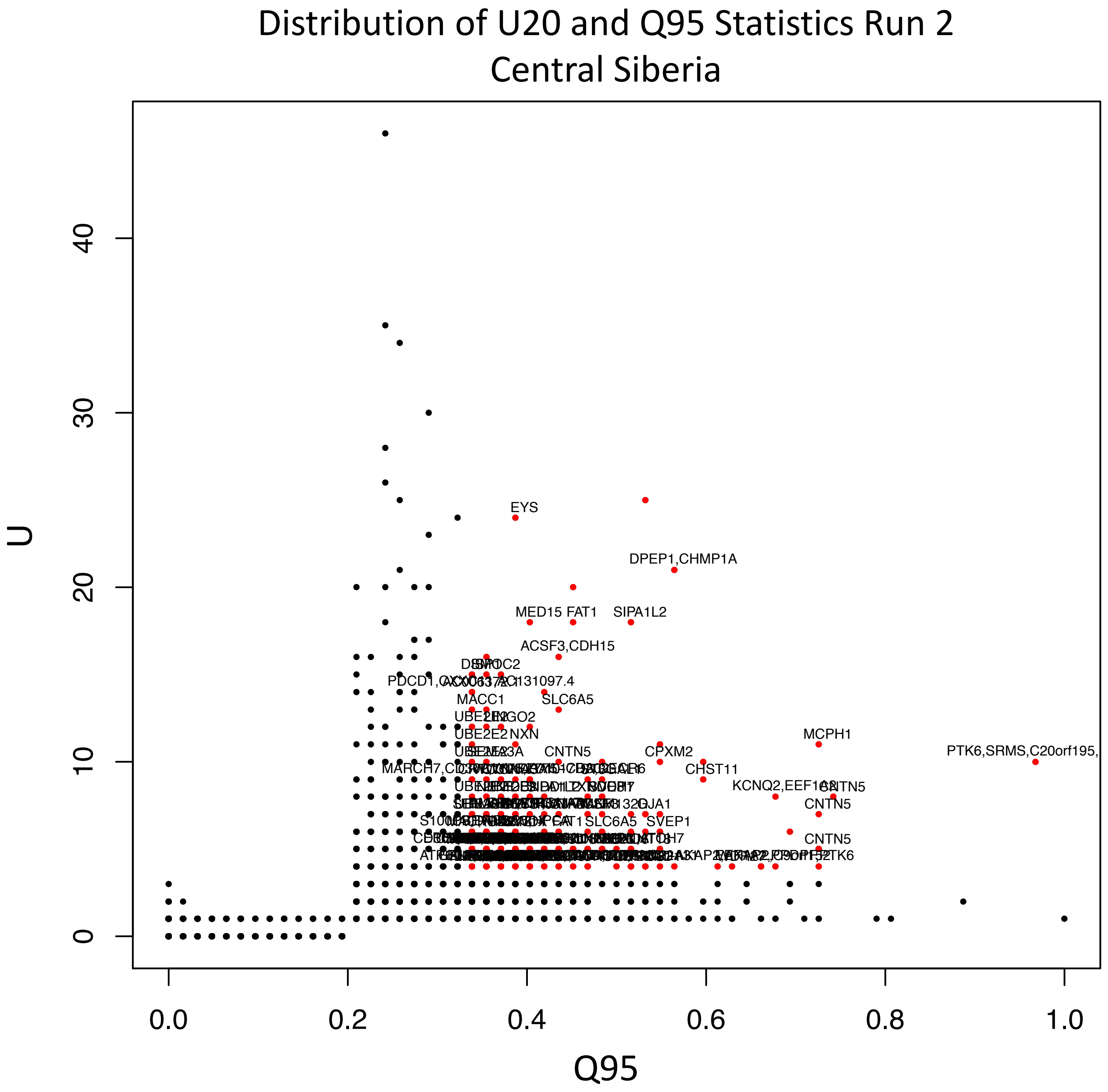

Distribution of U20 and Q95 Statistics Run 2
Central Siberia
U
Q95
0
10
20
30
40
0.0
0.2
0.4
0.6
0.8
1.0
EYS
DPEP1,CHMP1A
MED15
FAT1
SIPA1L2
ACSF3,CDH15
MACC1
SLC6A5
NXN
CNTN5
CPXM2
CHST11
MCPH1
PTK6,SRMS,C20orf195,
CNTN5
CNTN5
SLC6A5
SVEP1
CNTN5
PTK6

Figure S3

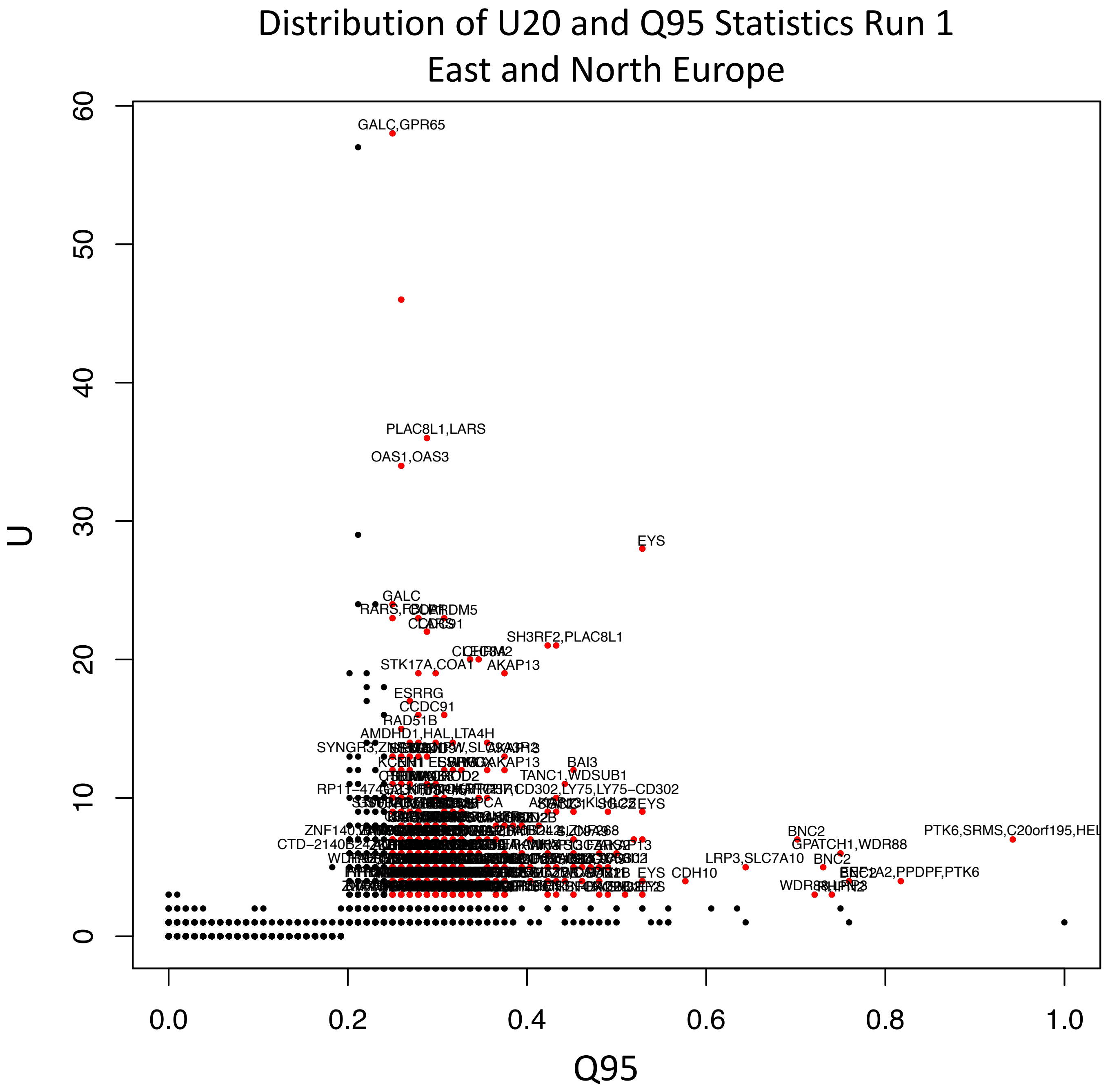
Distribution of U20 and Q95 Statistics Run 1
East and North Europe
U
Q95
0
10
20
30
40
50
60
0.0
0.2
0.4
0.6
0.8
1.0
GALC,GPR65
PLAC8L1,LARS
OAS1,OAS3
EYS
GALC
SH3RF2,PLAC8L1
STK17A,COA1
AKAP13
ESRRG
CCDC91
RAD51B
AMDHD1,HAL,LTA4H
BAI3
TANC1,WDSUB1
BNC2
PTK6,SRMS,C20orf195,HEL
GPATCH1,WDR88
LRP3,SLC7A10
BNC2
CDH10
EEF1A2,PPDPF,PTK6

Figure S4

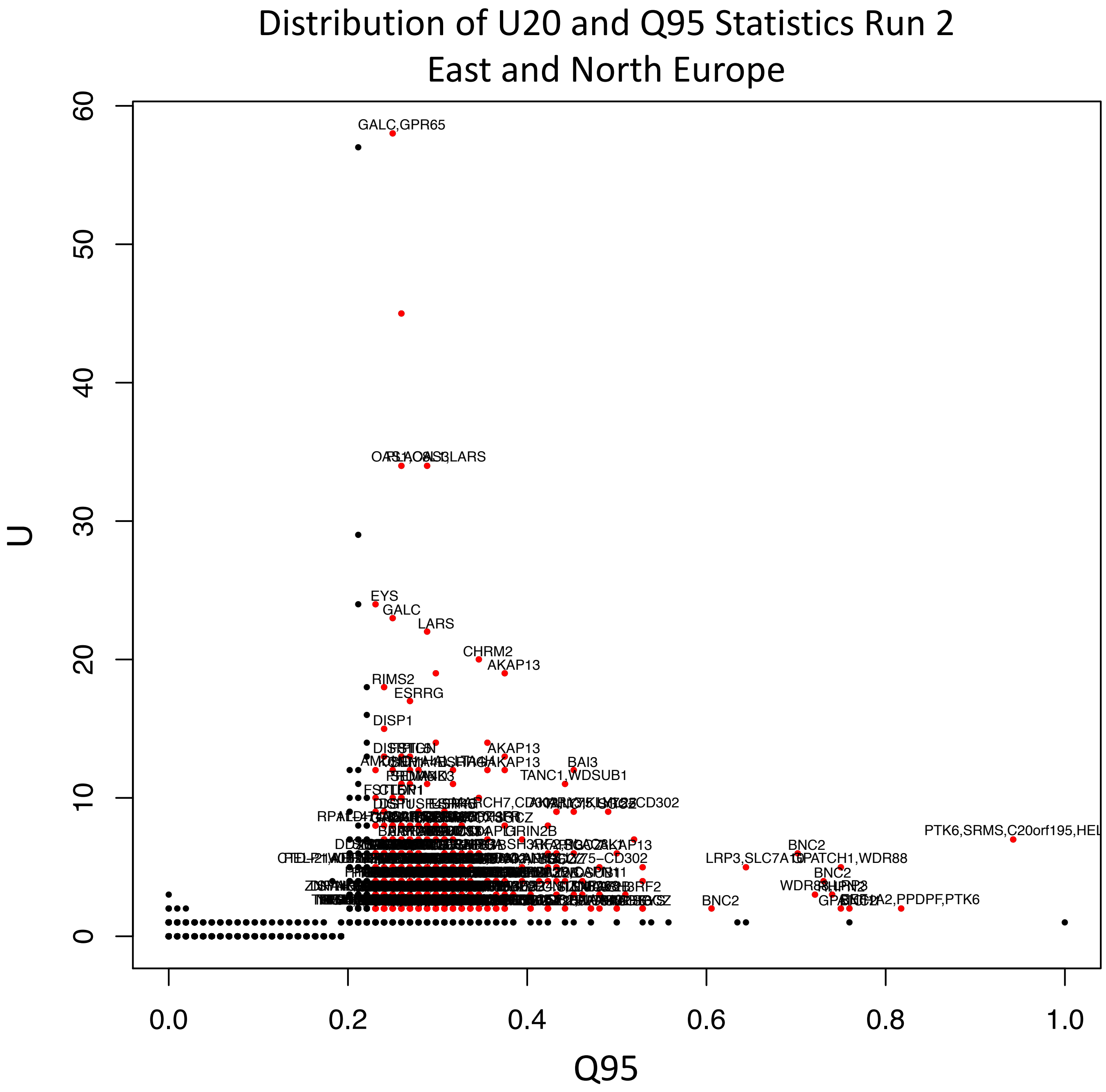
Distribution of U20 and Q95 Statistics Run 2
East and North Europe
U
Q95
0
10
20
30
40
50
60
0.0
0.2
0.4
0.6
0.8
1.0
GALC,GPR65
EYS
GALC
LARS
CHRM2
AKAP13
RIMS2
ESRRG
DISP1
AKAP13
BAI3
TANC1,WDSUB1
GRIN2B
BNC2
LRP3,SLC7A10
GPATCH1,WDR88
BNC2
BNC2
PTK6,SRMS,C20orf195,HEL

Figure S5

Distribution of U20 and Q95 Statistics Run 1
East and Southeast Asia Mainland

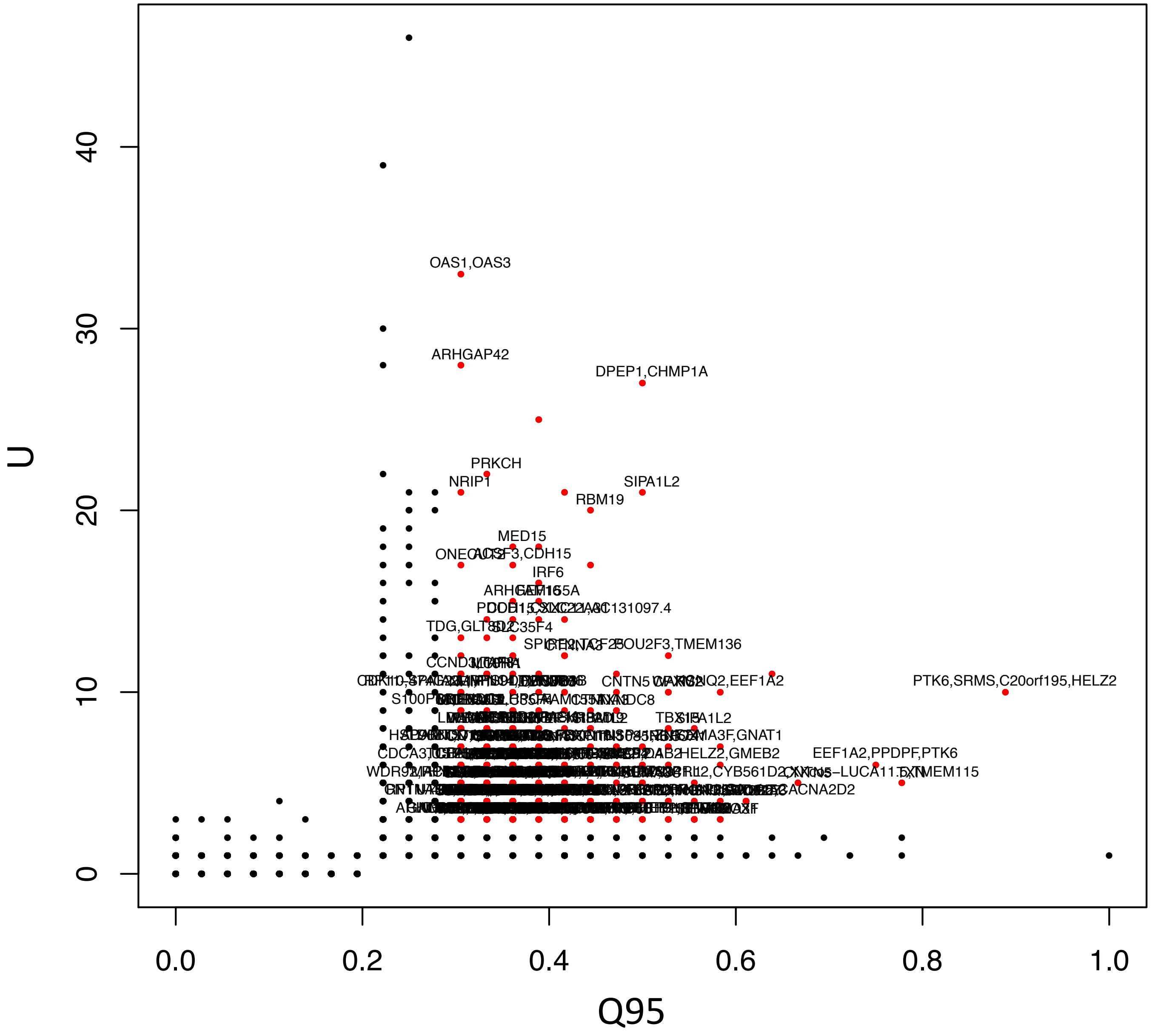

Figure S6

Distribution of U20 and Q95 Statistics Run 2
East and Southeast Asia Mainland

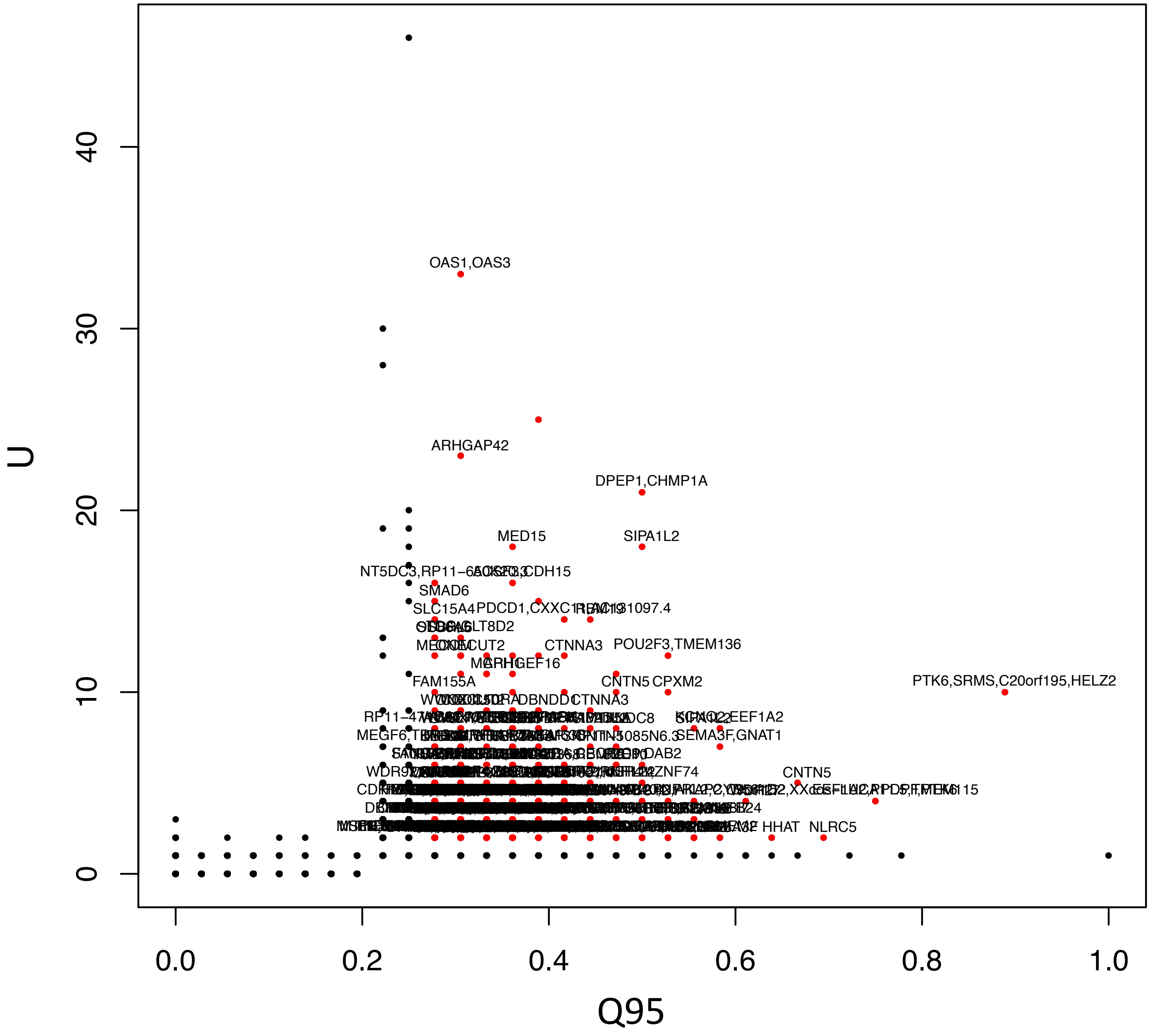

Figure S7

Distribution of U20 and Q95 Statistics Run 1
Island Southeast Asia

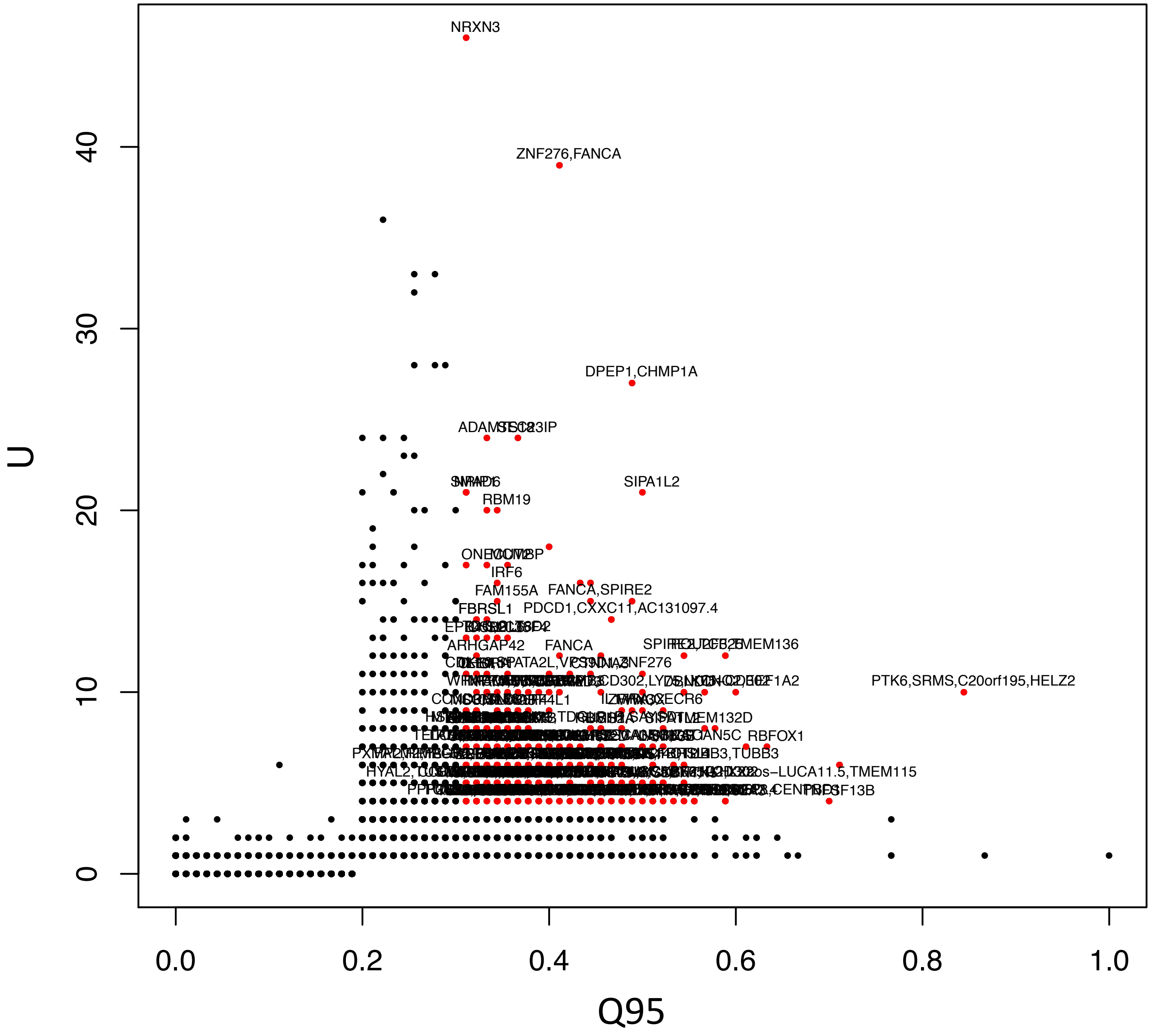

Figure S8

Distribution of U20 and Q95 Statistics Run 2
Island Southeast Asia

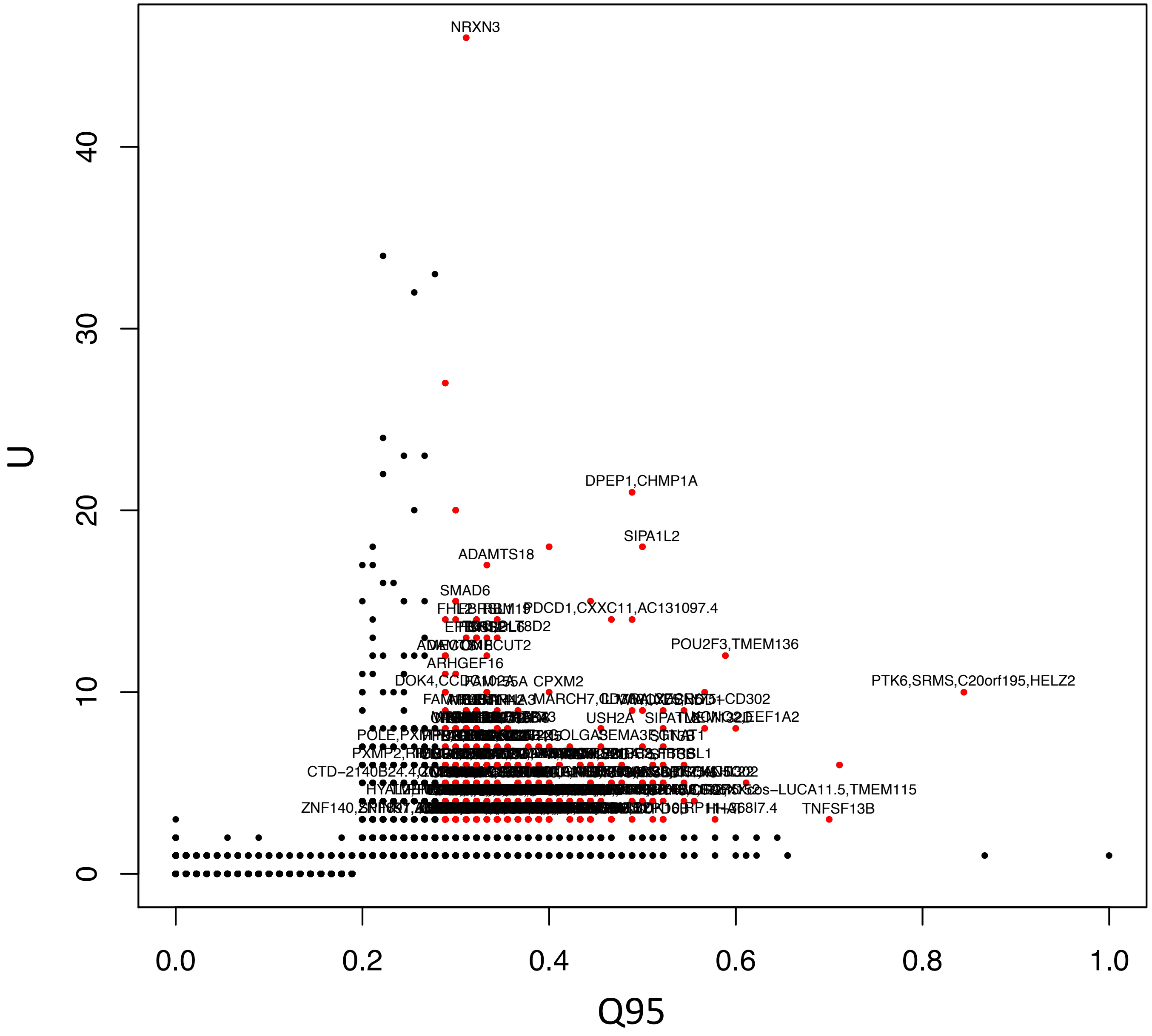

Figure S9

Distribution of U20 and Q95 Statistics Run 1
Northeast Siberia

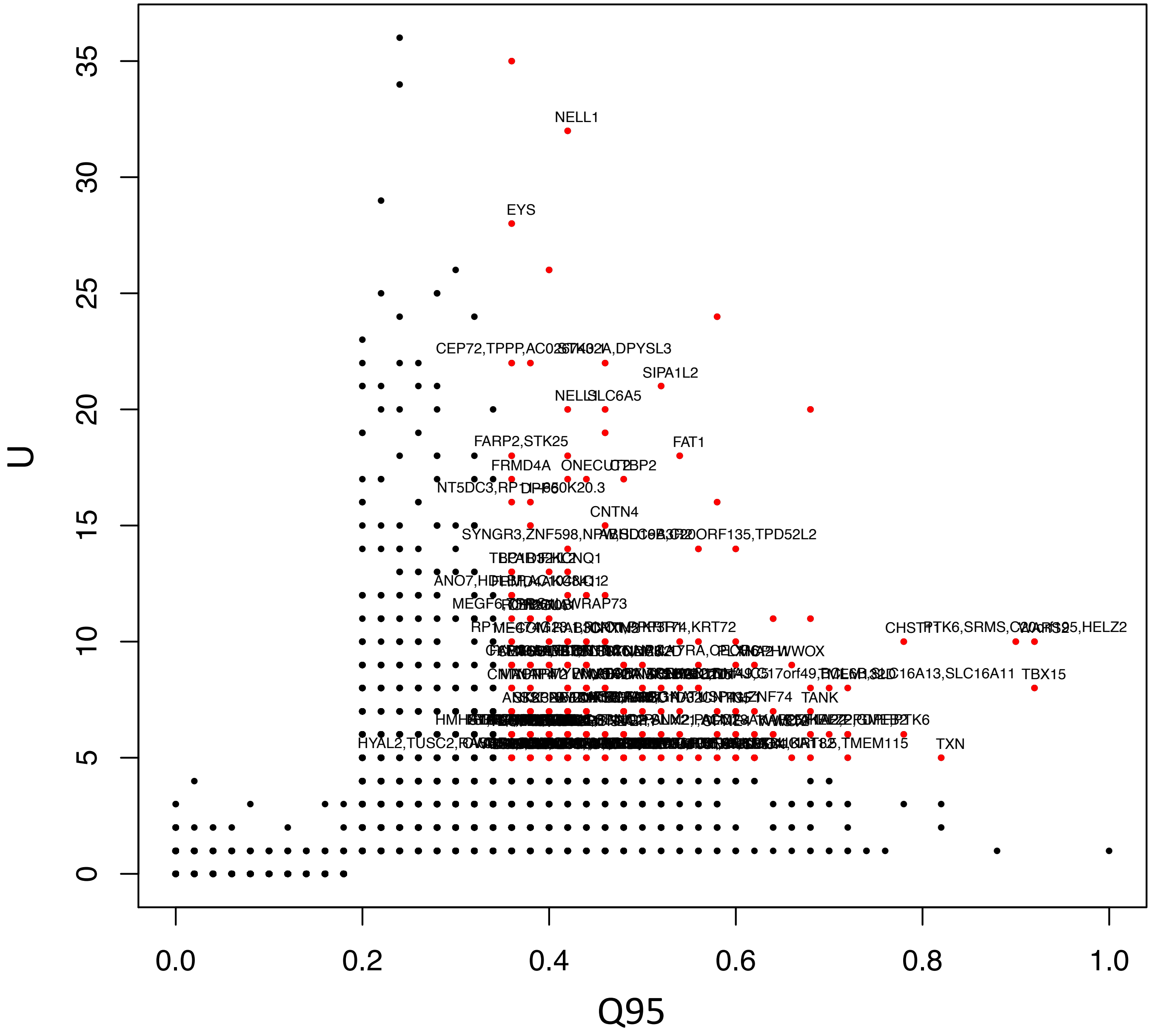

Figure S10

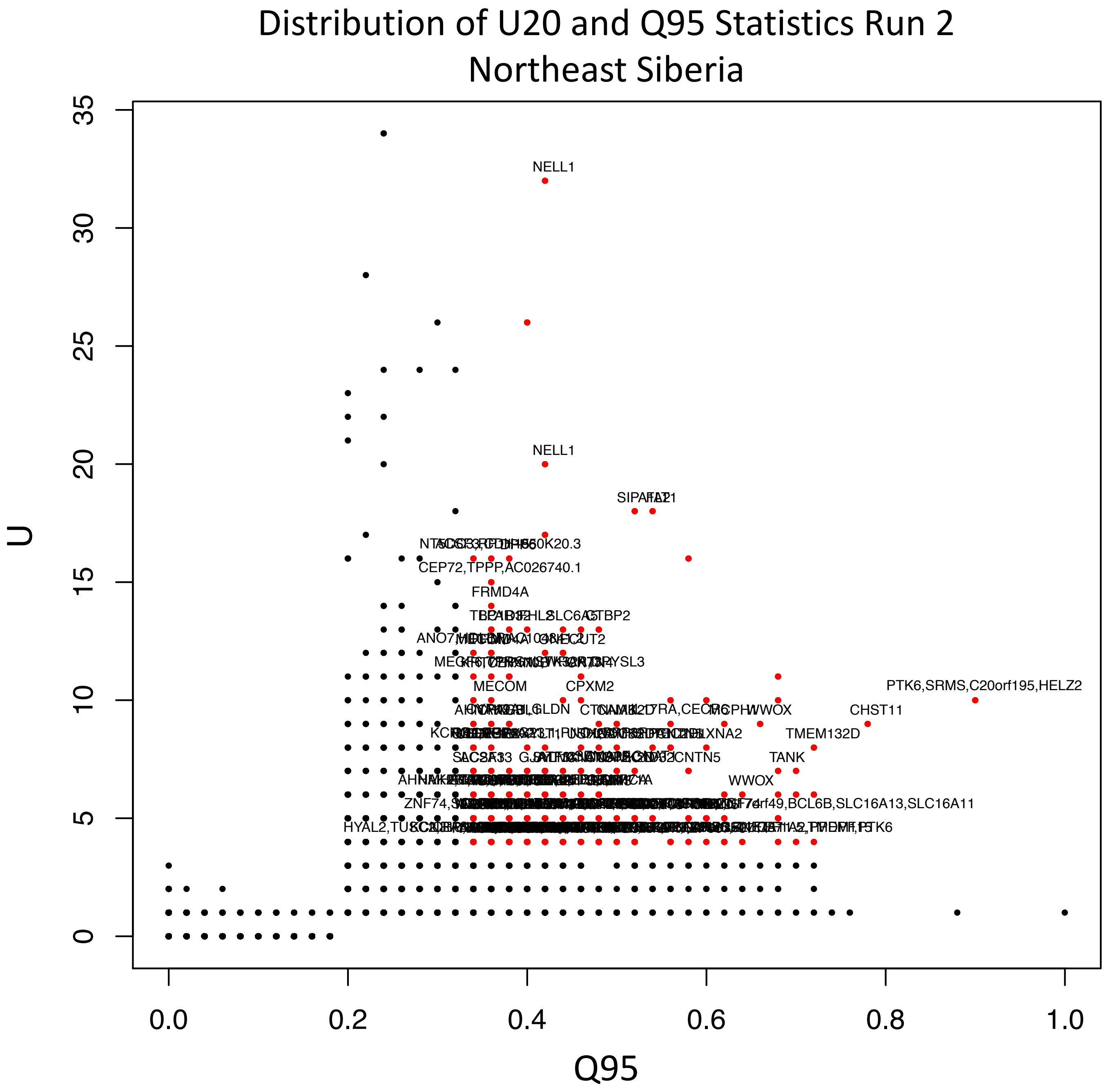
Distribution of U20 and Q95 Statistics Run 2
Northeast Siberia
NELL1
NELL1
CEP72,TPPP,AC026740.1
FRMD4A
MECOM
CPXM2
PTK6,SRMS,C20orf195,HELZ2
CHST11
TMEM132D
TANK
WWOX
U
Q95
0
5
10
15
20
25
30
35
0.0
0.2
0.4
0.6
0.8
1.0

Figure S11

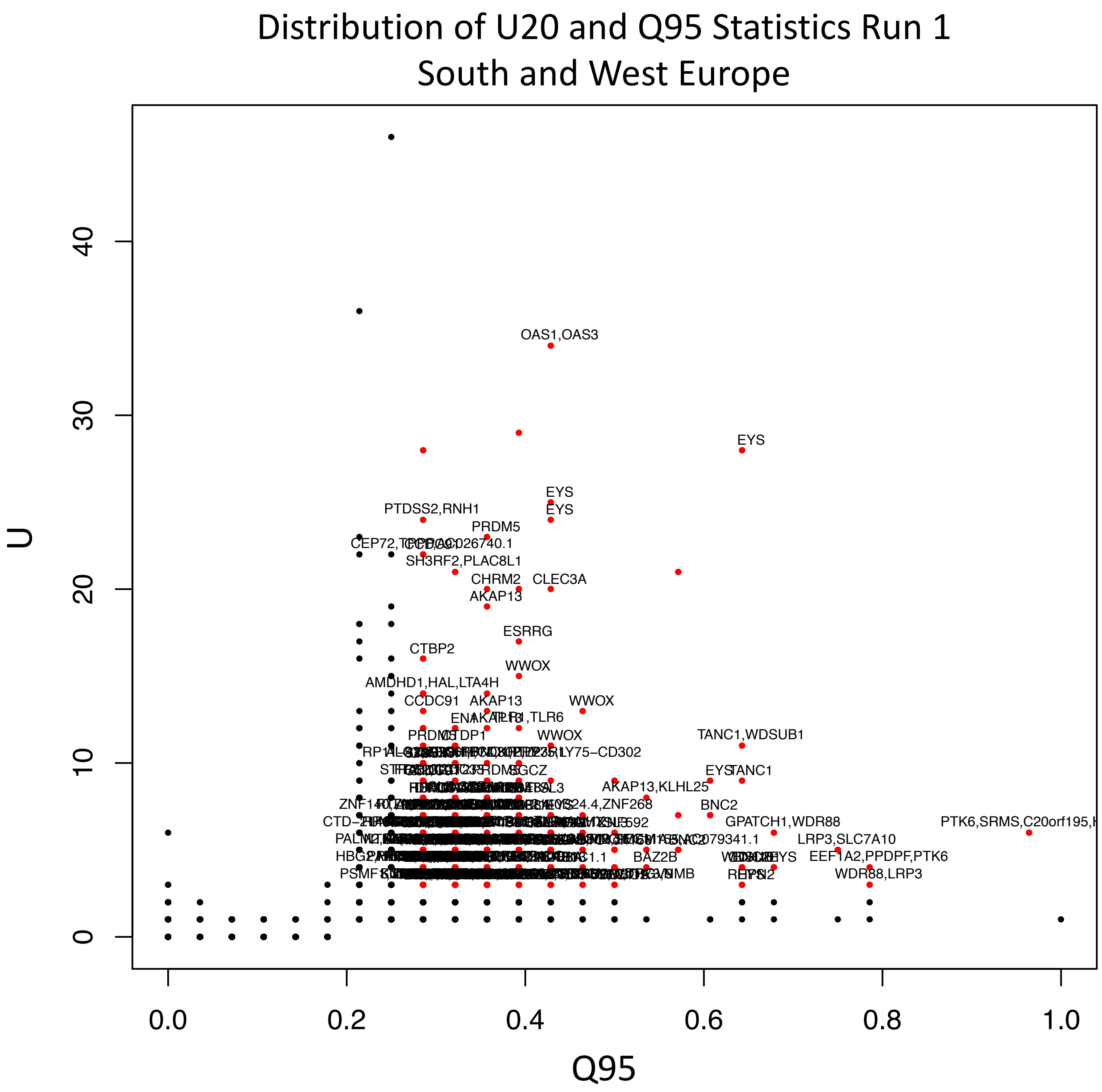
Distribution of U20 and Q95 Statistics Run 1
South and West Europe
U
Q95
0.0
0.2
0.4
0.6
0.8
1.0
0
10
20
30
40
OAS1,OAS3
EYS
PTDSS2,RNH1
PRDM5
SH3RF2,PLAC8L1
CHRM2
CLEC3A
AKAP13
ESRRG
CTBP2
WWOX
AMDHD1,HAL,LTA4H
CCDC91
WWOX
TANC1,WDSUB1
TANC1
AKAP13,KLHL25
BNC2
GPATCH1,WDR88
LRP3,SLC7A10
EEF1A2,PPDPF,PTK6
WDR88,LRP3
BAZ2B

Figure S12

Distribution of U20 and Q95 Statistics Run 2
South and West Europe

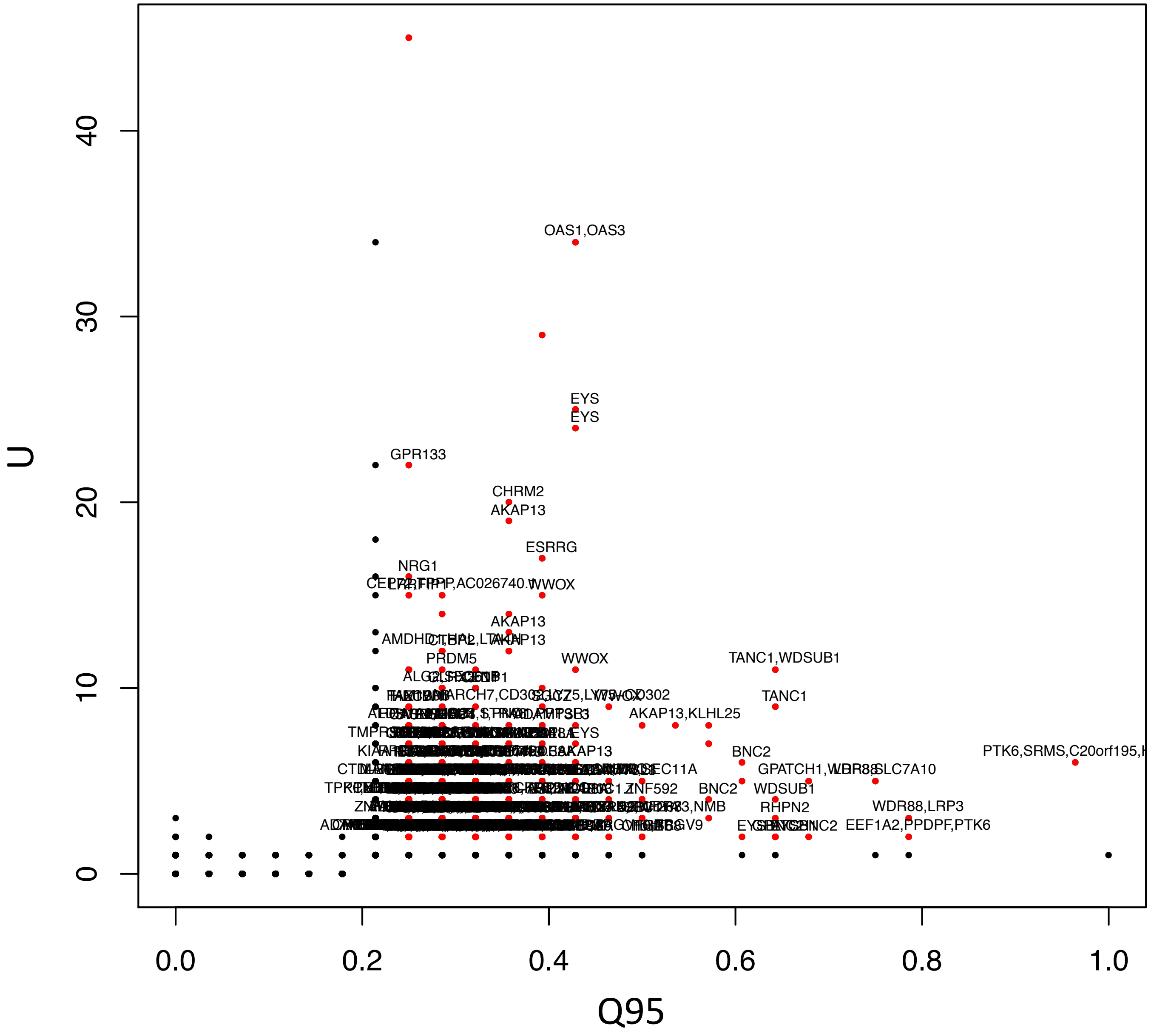

Figure S13

Distribution of U20 and Q95 Statistics Run 1
South Asia

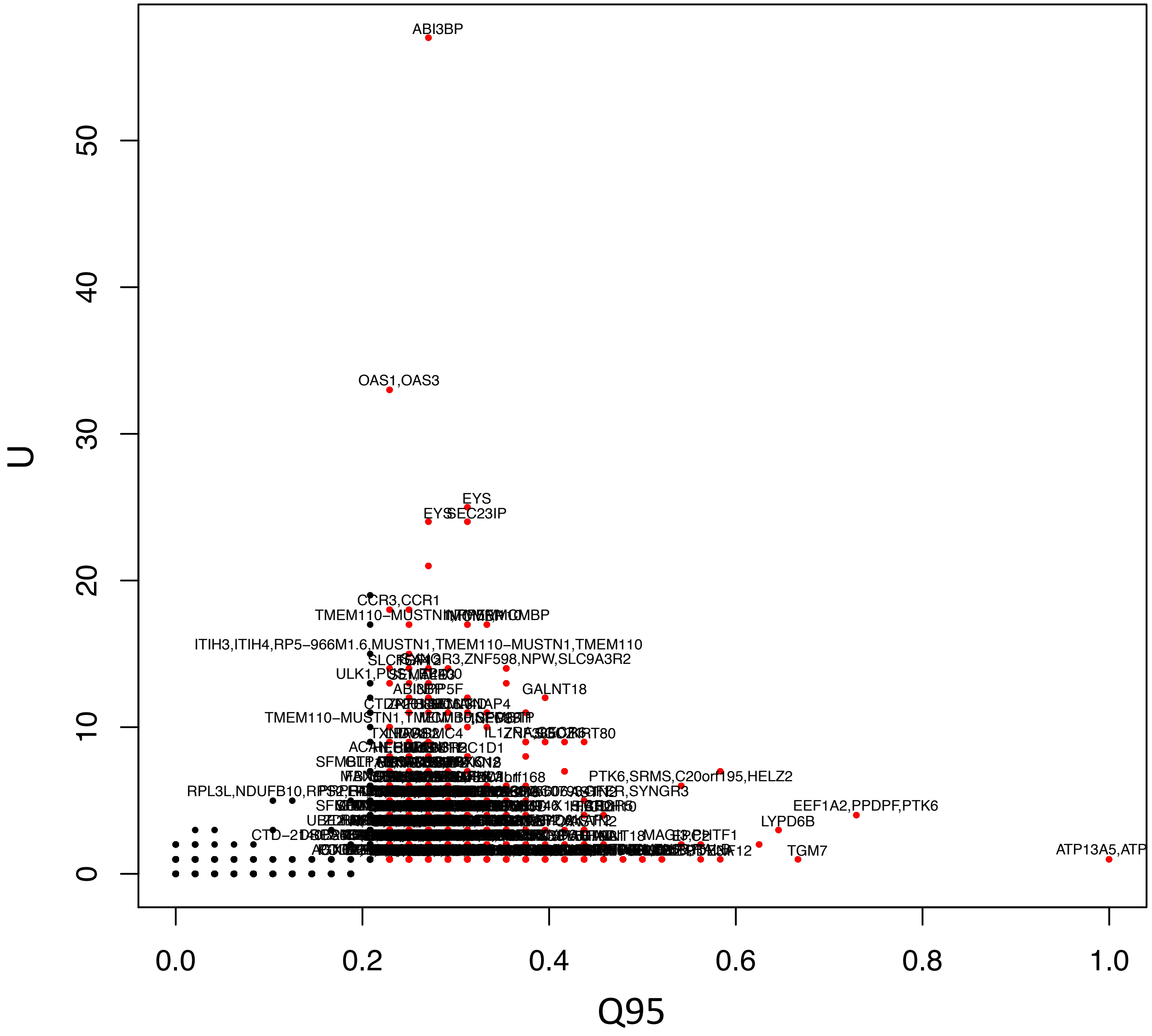

Figure S14

Distribution of U20 and Q95 Statistics Run 2
South Asia

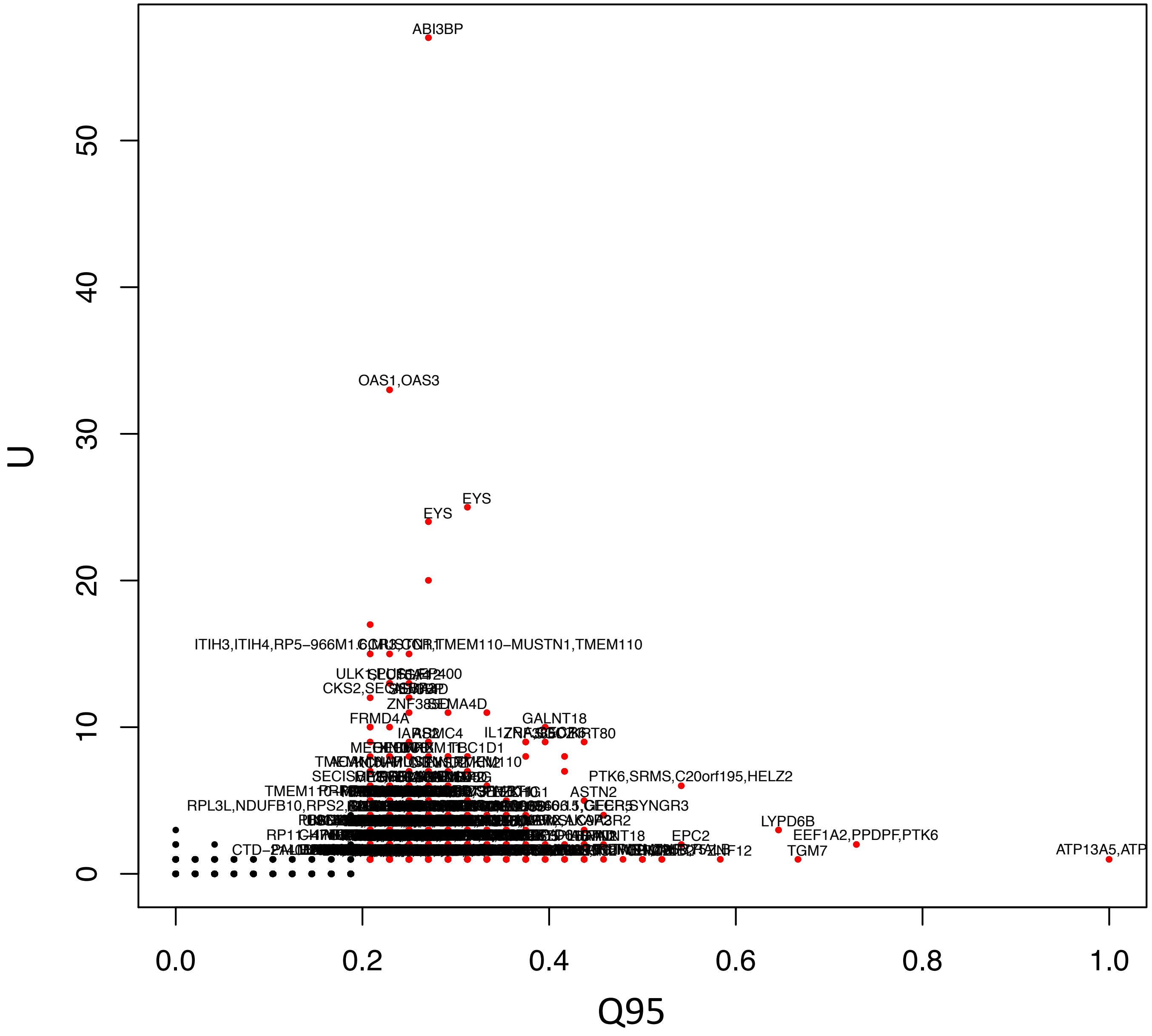

Supplement: Supplementary Data [file msx314_supp.zip › Supplementary Figures 1-14_Final.pdf]
