# Supplementary material for: Disentangling Immediate Adaptive Introgression from Selection on Standing Introgressed Variation in Humans
Source: Mol Biol Evol. 2017 Dec 6;35(3):623–30. doi: 10.1093/molbev/msx314 (PMC5850494; doi:10.1093/molbev/msx314)

Figure S15

Distribution of U20 and Q95 Statistics Run 1
South Siberia and Mongolia

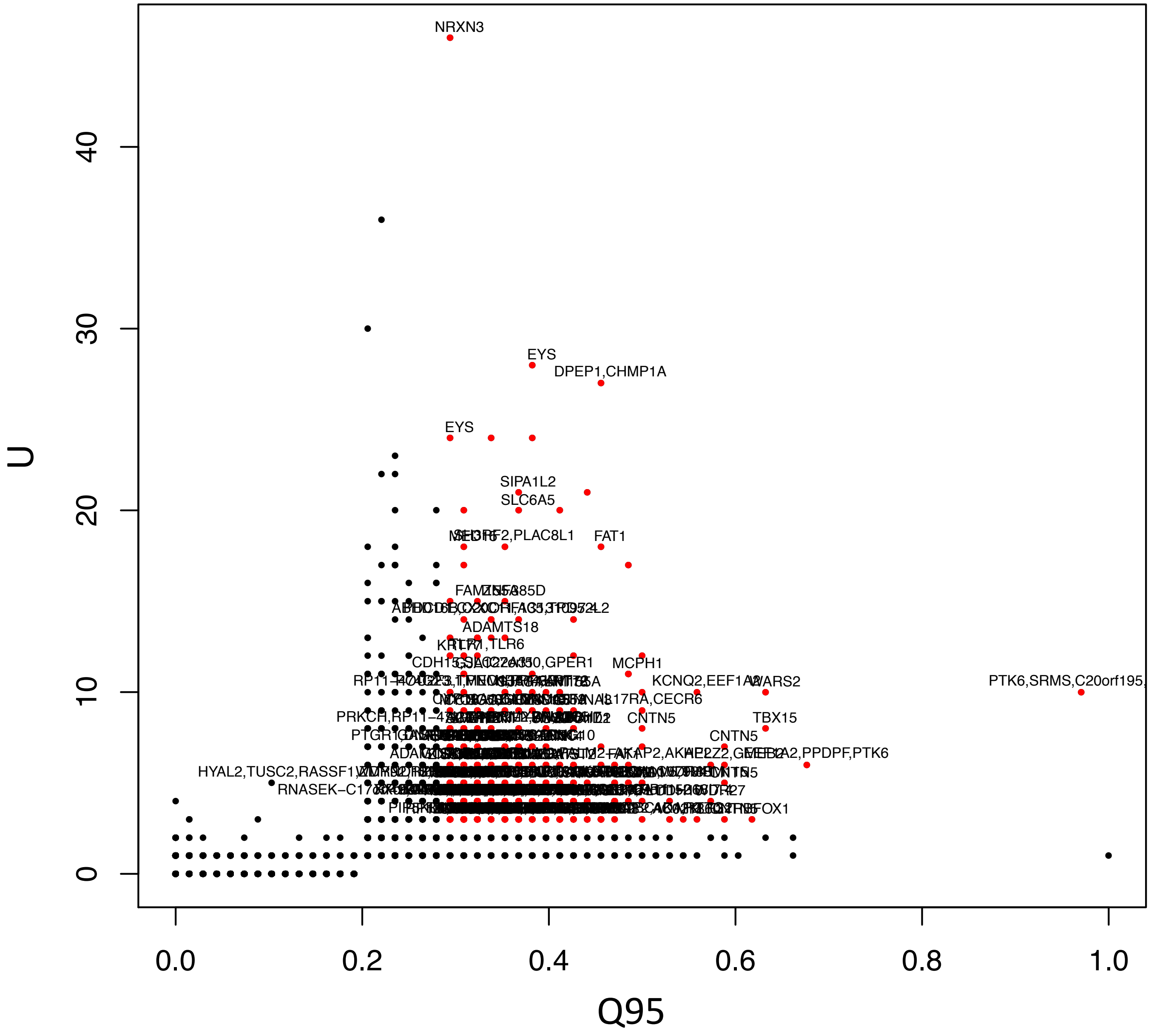

Figure S16

Distribution of U20 and Q95 Statistics Run 2
South Siberia and Mongolia

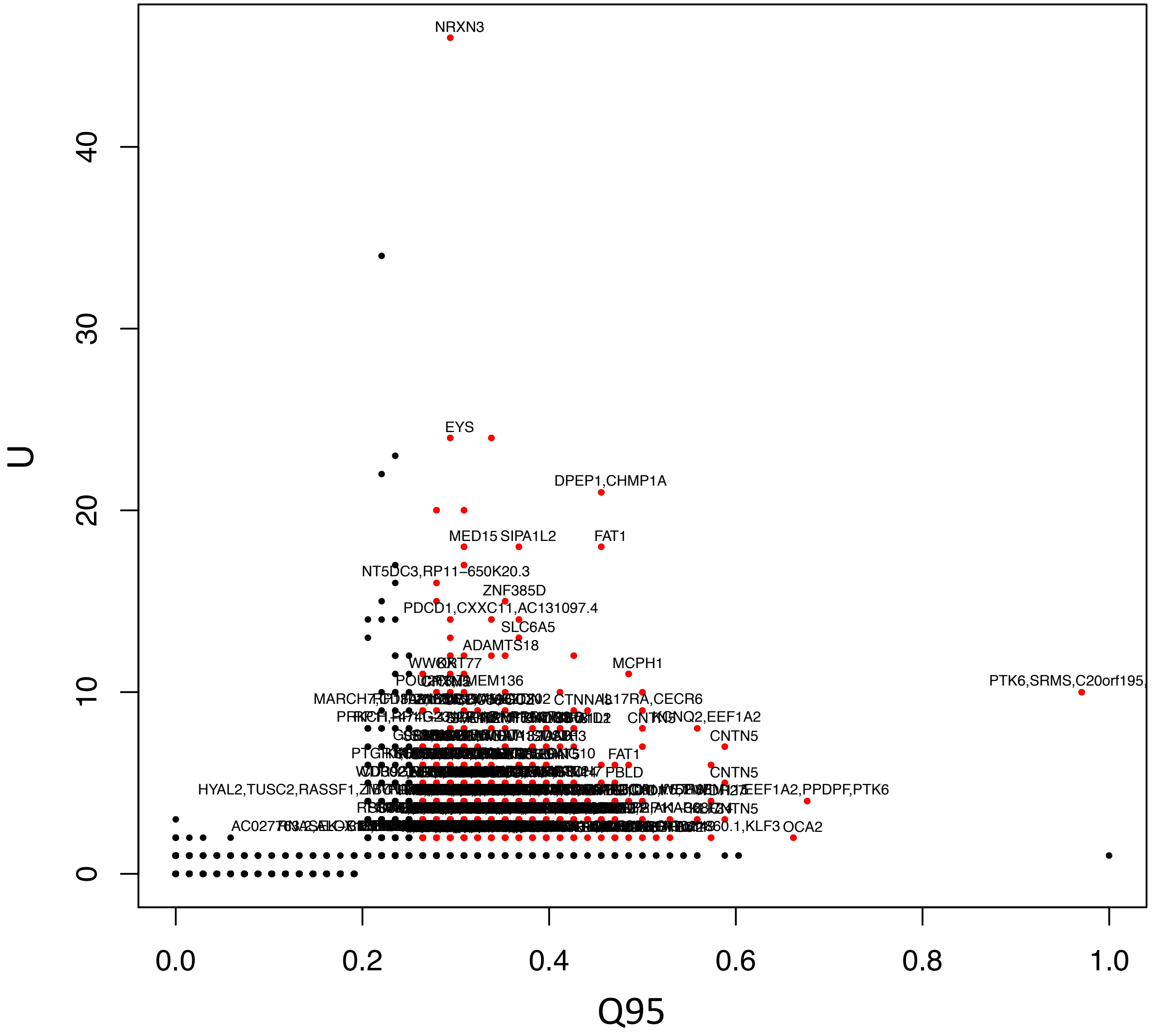

Figure S17

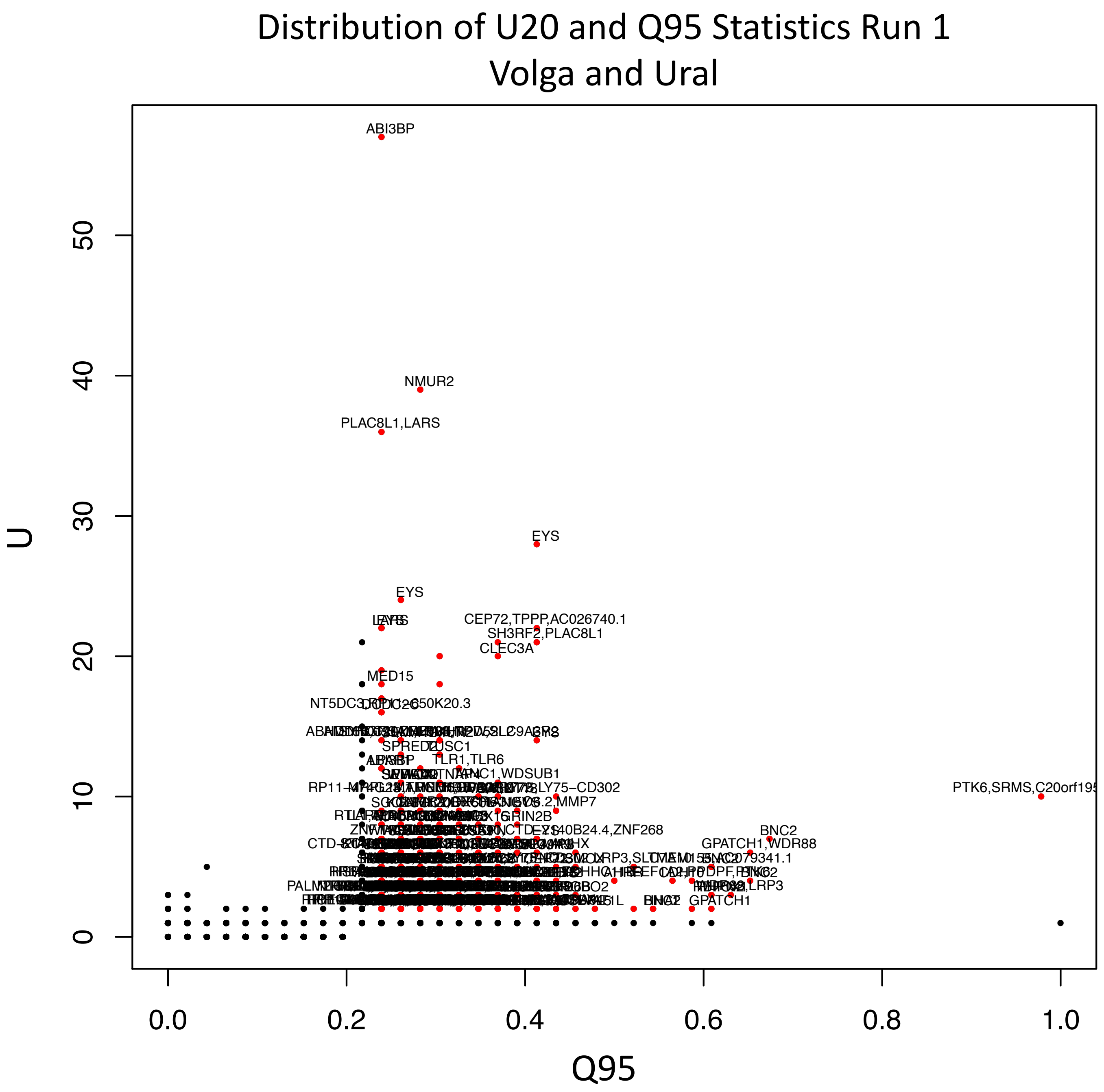
Distribution of U20 and Q95 Statistics Run 1
Volga and Ural
U
Q95
0.0
0.2
0.4
0.6
0.8
1.0
0
10
20
30
40
50
ABI3BP
NMUR2
PLAC8L1,LARS
EYS
EYS
CEP72,TPPP,AC026740.1
SH3RF2,PLAC8L1
CLEC3A
MED15
TLR1,TLR6
PTK6,SRMS,C20orf195
BNC2
GPATCH1,WDR88
GPATCH1

Figure S18

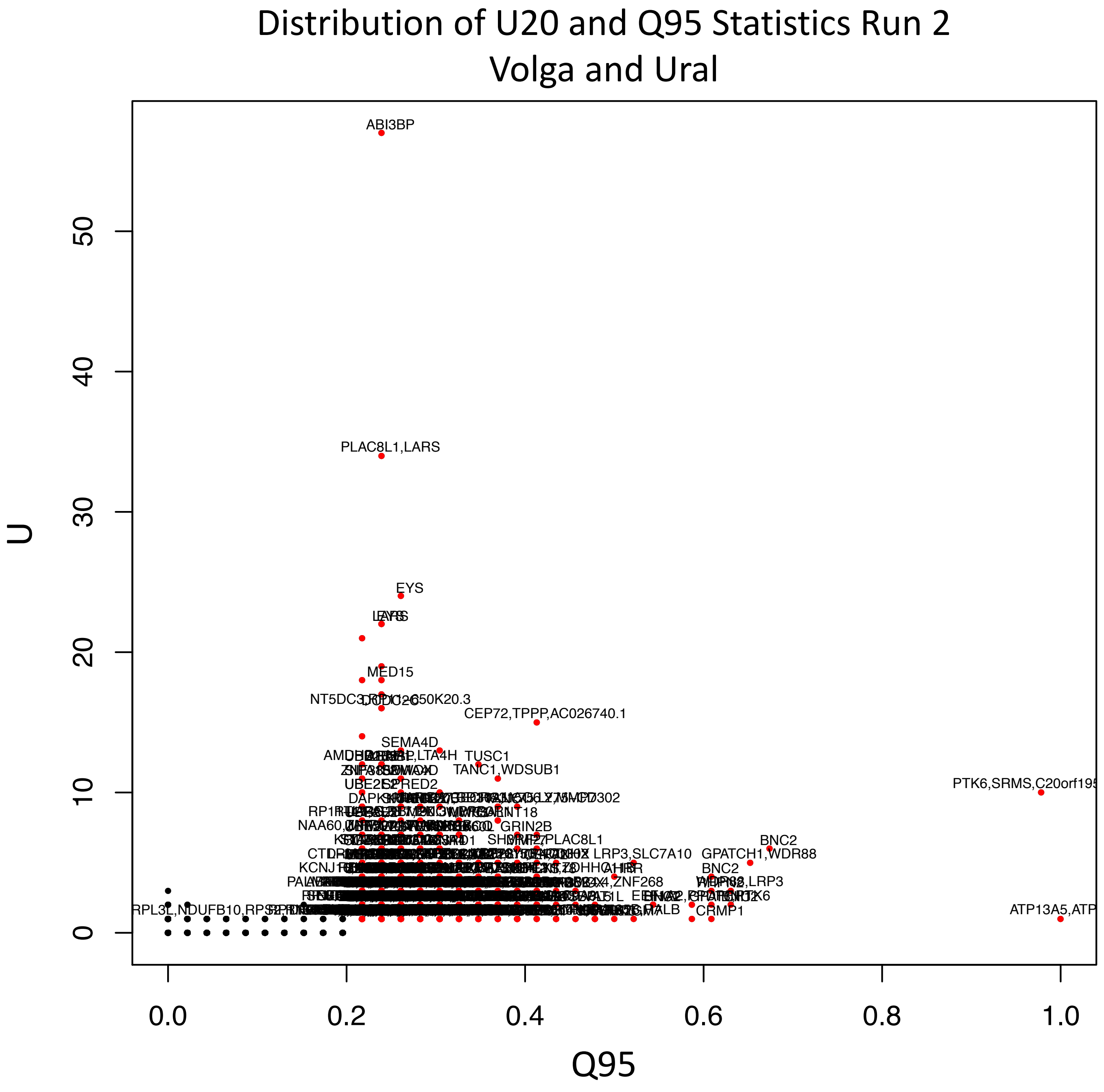
Distribution of U20 and Q95 Statistics Run 2
Volga and Ural
U
Q95
0
10
20
30
40
50
0.0
0.2
0.4
0.6
0.8
1.0
ABI3BP
PLAC8L1,LARS
EYS
MED15
CEP72,TPPP,AC026740.1
SEMA4D
TUSC1
TANC1,WDSUB1
GRIN2B
BNC2
GPATCH1,WDR88
LRP3,SLC7A10
CRMP1
PTK6,SRMS,C20orf195
ATP13A5,ATP

Figure S19

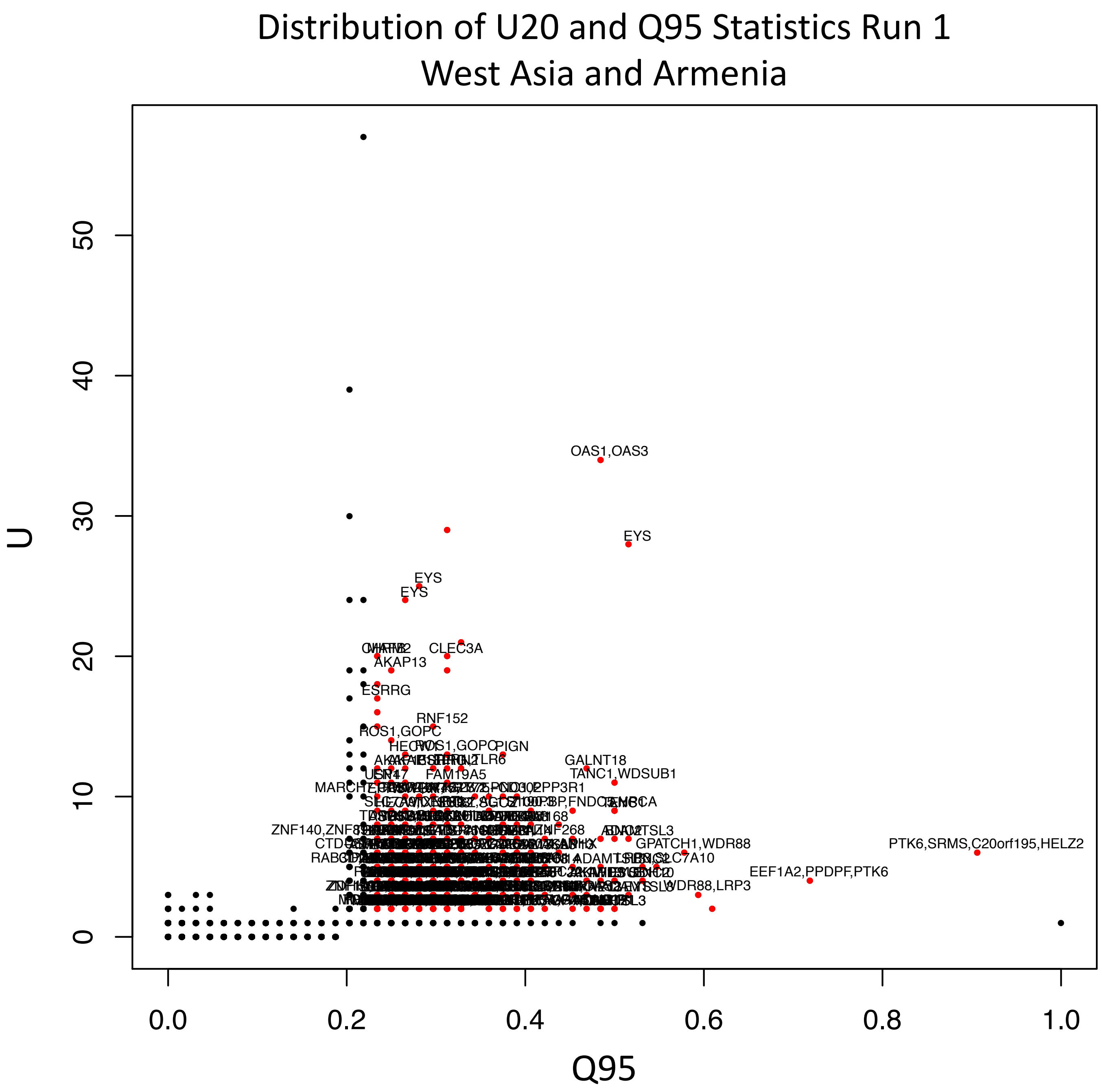
Distribution of U20 and Q95 Statistics Run 1
West Asia and Armenia
U
Q95
OAS1,OAS3
EYS
EYS
EYS
CLEC3A
AKAP13
ESRRG
RNF152
ROS1,GOPC
PIGN
GALNT18
TANC1,WDSUB1
FAM19A5
GPATCH1,WDR88
PTK6,SRMS,C20orf195,HELZ2
EEF1A2,PPDPF,PTK6
WDR88,LRP3
0.0
0.2
0.4
0.6
0.8
1.0
0
10
20
30
40
50

Figure S20

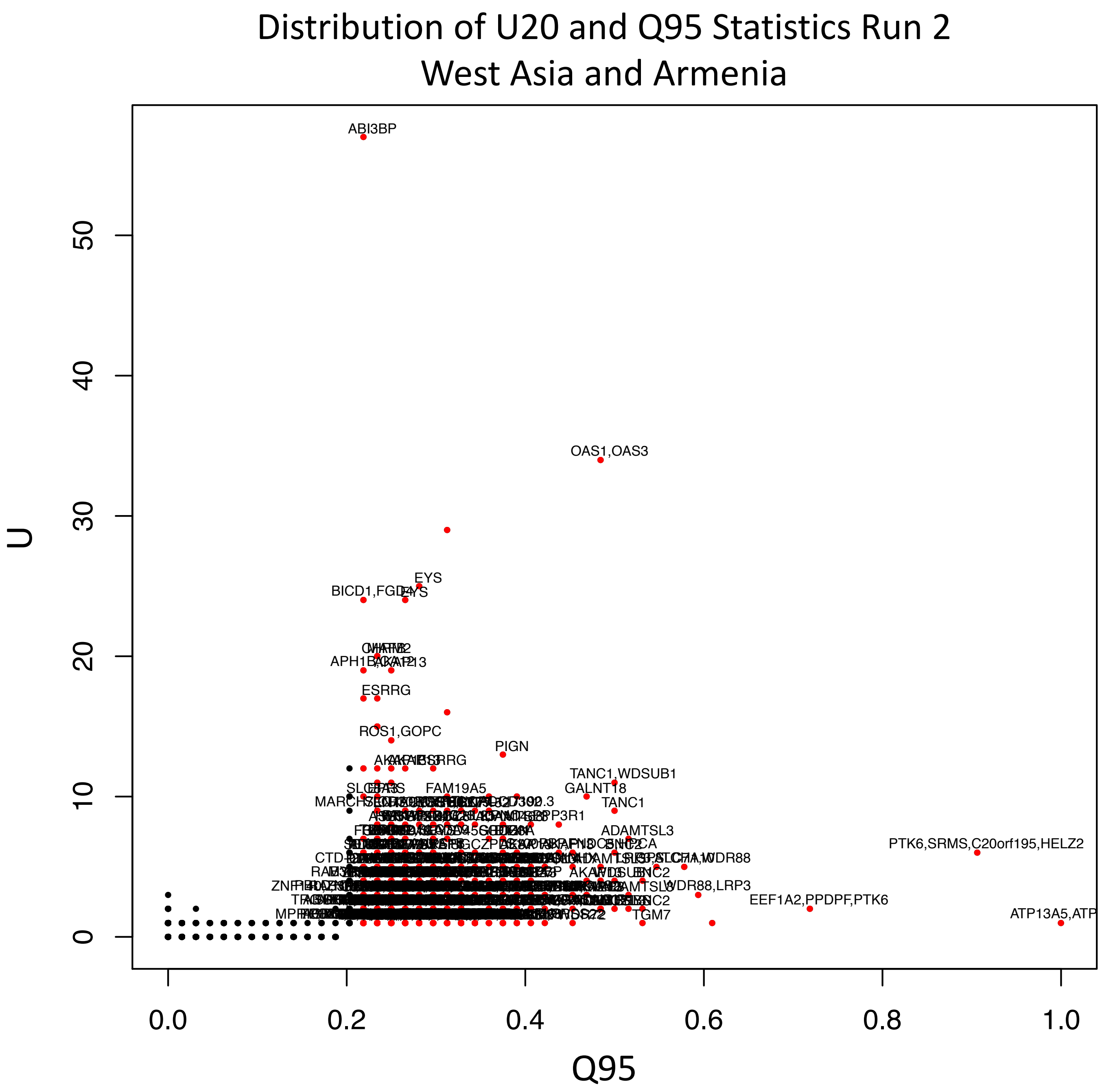
Distribution of U20 and Q95 Statistics Run 2
West Asia and Armenia
U
Q95
0
10
20
30
40
50
0.0
0.2
0.4
0.6
0.8
1.0
ABI3BP
OAS1,OAS3
EYS
ESRRG
ROS1,GOPC
PIGN
TANC1,WDSUB1
GALNT18
TANC1
FAM19A5
ADAMTSL3
PTK6,SRMS,C20orf195,HELZ2
WDR88,LRP3
EEF1A2,PPDPF,PTK6
ATP13A5,ATP
TGM7

Figure S21

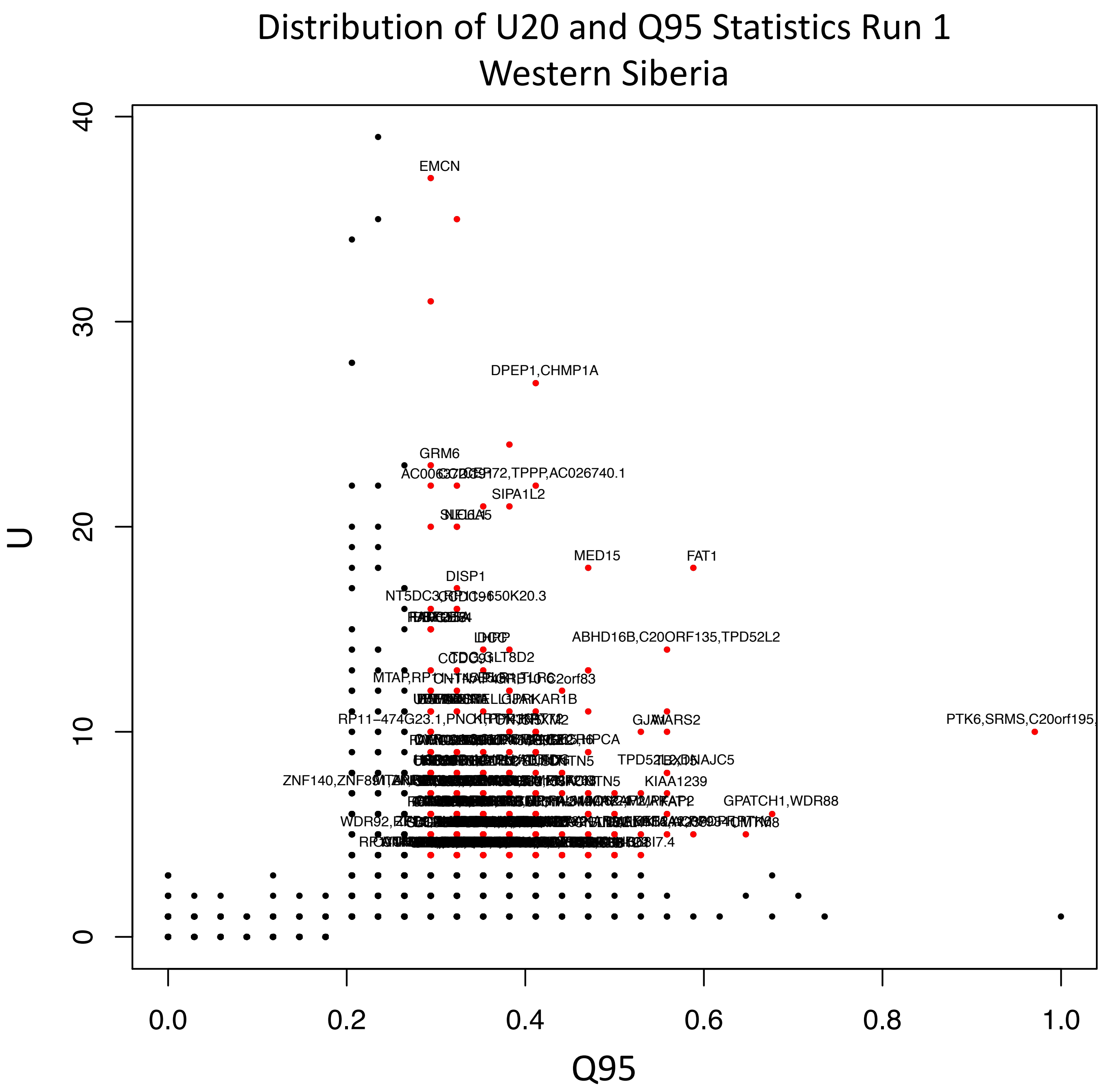
Distribution of U20 and Q95 Statistics Run 1
Western Siberia
EMCN
DPEP1,CHMP1A
GRM6
SIPA1L2
MED15
FAT1
DISP1
ABHD16B,C20ORF135,TPD52L2
PTK6,SRMS,C20orf195,
KIAA1239
GPATCH1,WDR88
U
Q95
0
10
20
30
40
0.0
0.2
0.4
0.6
0.8
1.0

Figure S22

Distribution of U20 and Q95 Statistics Run 2
Western Siberia

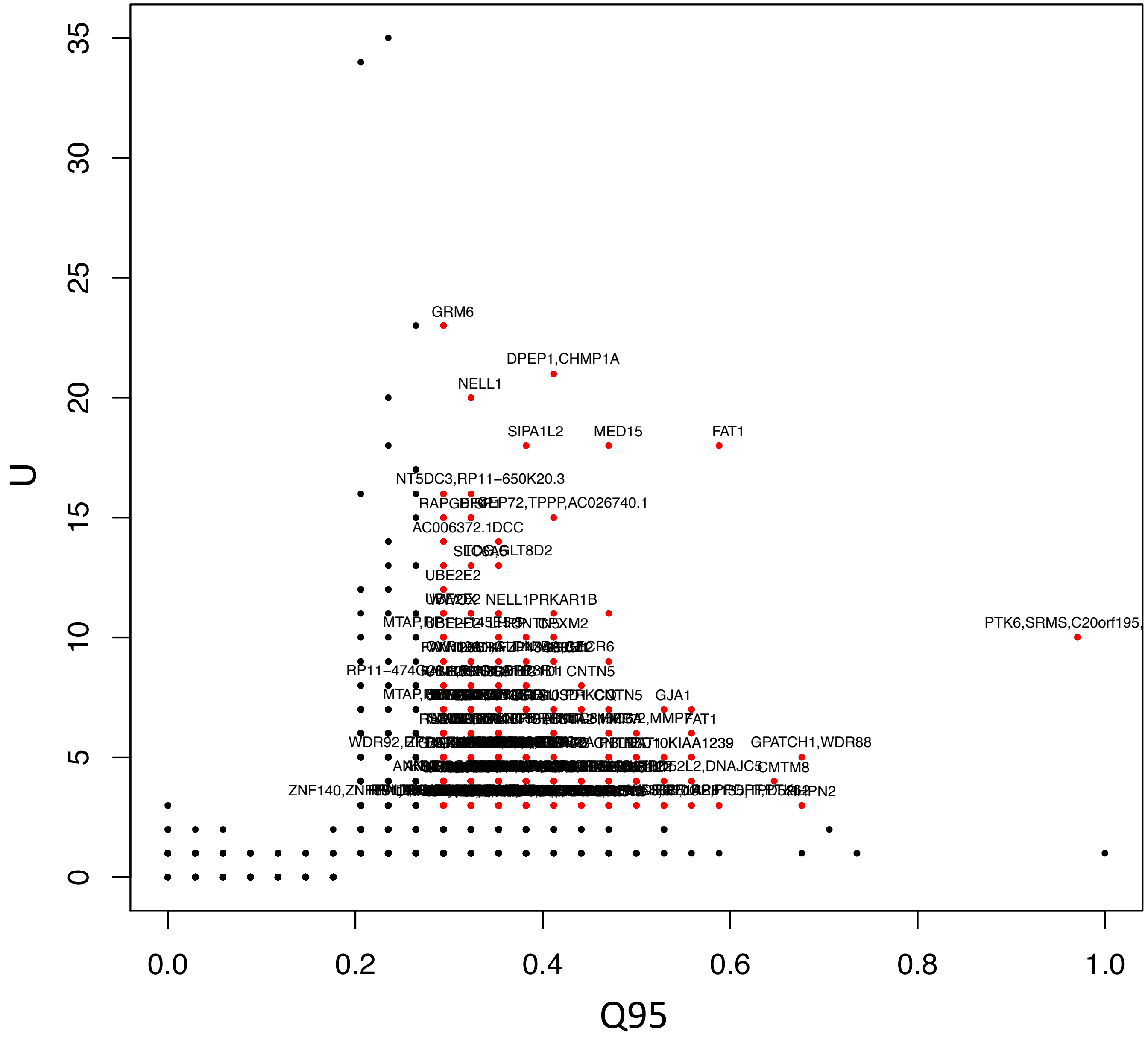

Supplement: Supplementary Data [file msx314_supp.zip › Supplementary Figures 15-22_Final.pdf]
