# Supplementary figures and images for: Disentangling Immediate Adaptive Introgression from Selection on Standing Introgressed Variation in Humans
Source: Mol Biol Evol. 2017 Dec 6;35(3):623–30. doi: 10.1093/molbev/msx314 (PMC5850494; doi:10.1093/molbev/msx314)

Figure S23

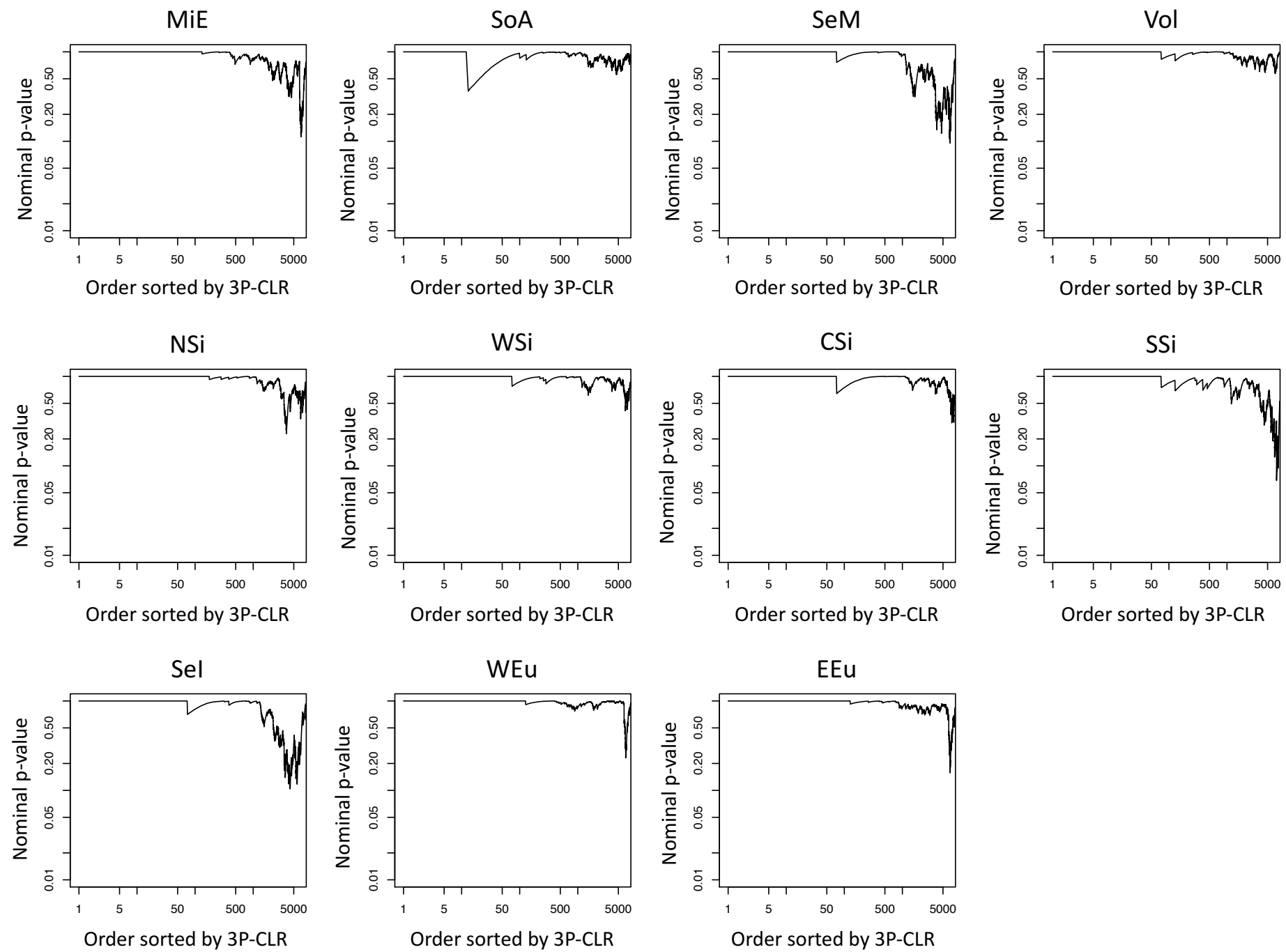

Figure S24

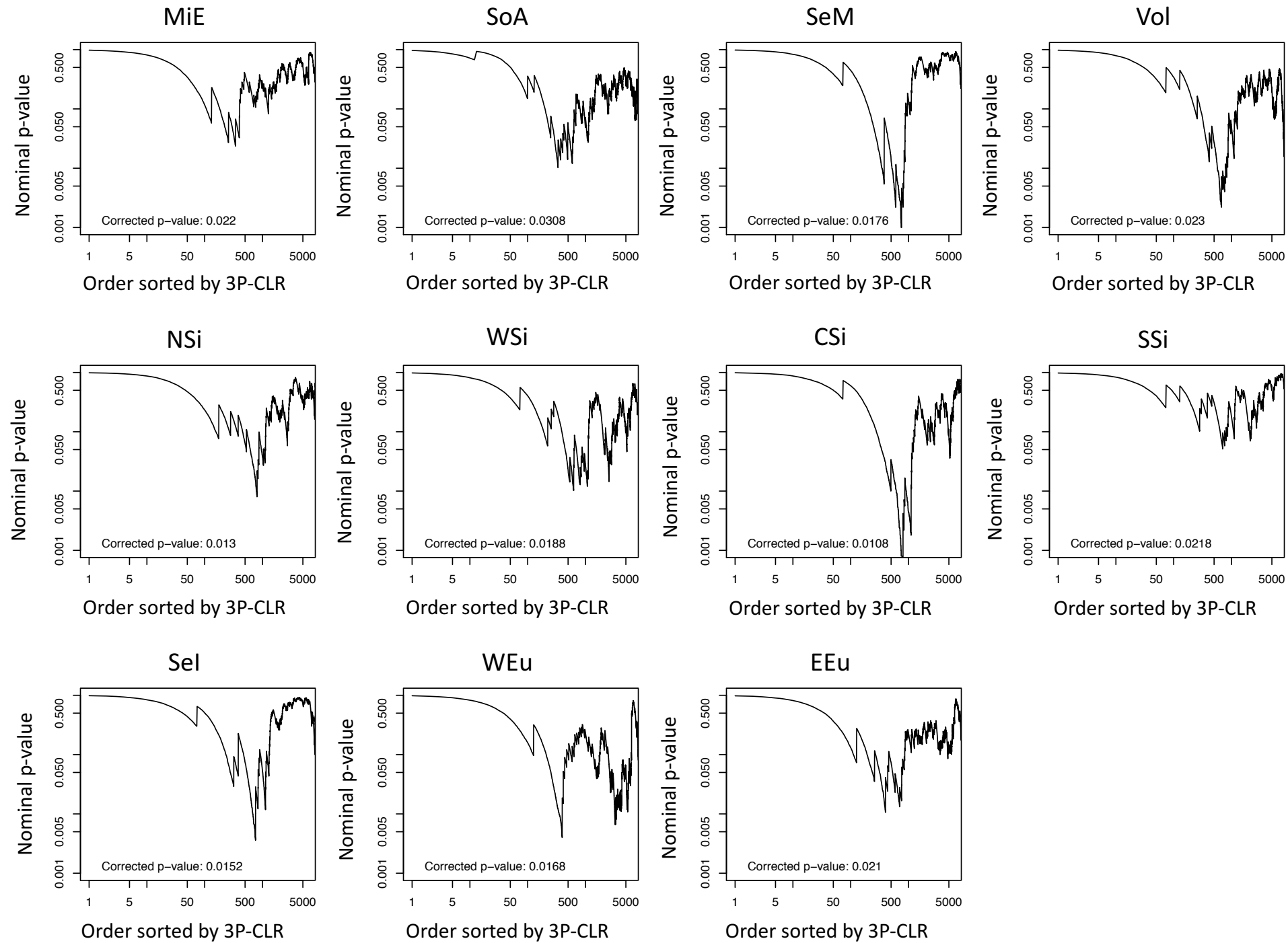

Figure S25

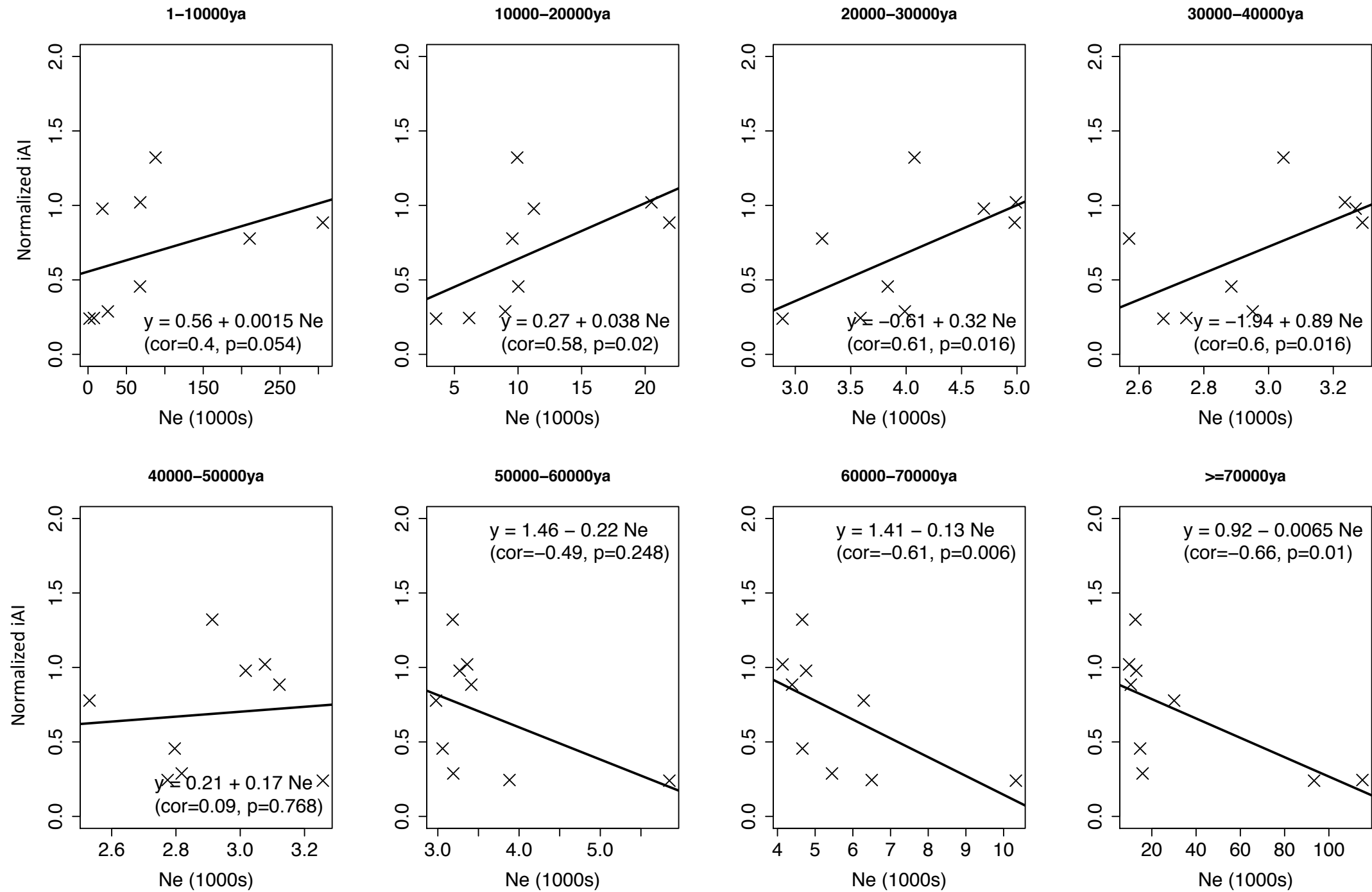

Figure S26

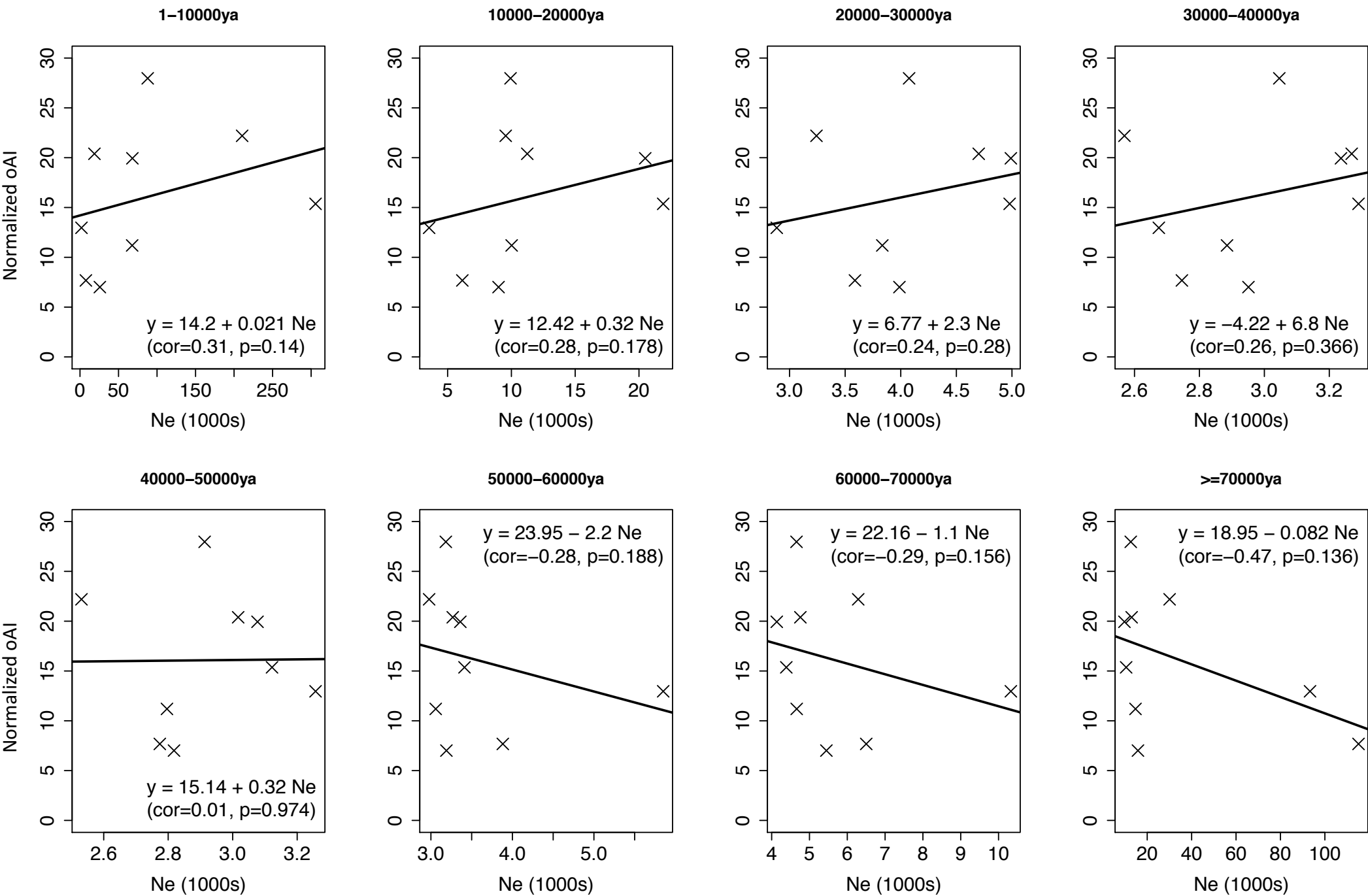

Supplement: Supplementary Data [file msx314_supp.zip › Supplementary Figures Revision 23_26_Final.pdf]
